# The comparative analysis of Sino-Japanese football policies from the perspective of policy instruments

**DOI:** 10.1371/journal.pone.0354667

**Published:** 2026-07-29

**Authors:** Chuanmin Zhang, Zanying Hu, Huqiang Wei, Mei Peng, Fei Gao

**Affiliations:** School of Physical Education, Tianjin University of Sport, Tianjin, China; Universidade de Aveiro Escola Superior de Saude de Aveiro, PORTUGAL

## Abstract

Football development depends heavily on strong policy support, rendering policy analysis and evaluation of significant practical importance. This study uses Nvivo11 software and the PMC index model to conduct a quantitative comparative analysis of 31 Sino-Japanese football policy texts, focusing on policy instruments, content characteristics, and overall quality. The findings reveal that both countries prioritize football development, social participation, and club construction; China emphasizes campus football, while Japan focuses on elite player development and league performance. PMC evaluation indicates that Chinese policies score higher in system design and institutional capacity, whereas Japan performs better in implementation clarity and community linkage. Both countries share shortcomings in insufficient policy clarity and vague long-term competitive goals. The research results provide empirical evidence for the optimization of Chinese football policies and a reference for drawing on international experience.

## 1. Introduction

Football development cannot do without policy support, so policy analysis and evaluation are of vital importance. This study adopts Nvivo11 and the PMC index model to conduct a comparative analysis of Sino-Japanese football policies, exploring the application of policy instruments and differences in policy quality. As General Secretary Xi Jinping emphasized, “China’s modern football still lags considerably behind football powerhouses in terms of both popularization and competitive levels. We must concentrate our efforts on formulating an overarching plan for football reform and development and steadfastly advance the reform [1]” General Secretary Xi Jinping’s important expositions on football have set forth requirements and charted the course for Chinese football development. They contain two implications: firstly, enhancing comparative research on football policies with other countries to learn from their strengths and compensate for China’s weaknesses, in a bid to promote its high-quality development; secondly, leveraging China’s institutional advantages to deepen football reform and development. Formulating scientific and reasonable policies is paramount in this endeavor. In 2015, the General Office of the State Council issued the *Overall Plan for Chinese Football Reform and Development* [2] (hereinafter referred to as *The Plan*), which mapped out a blueprint for Chinese football development. Subsequently, four ministries and commissions, including the National Development and Reform Commission, jointly issued the *Medium-and Long-term Development Plan for Chinese Football (2016–2050)* [3], established medium-and long-term objectives for its development. In addition, relevant policies to promote the development of Chinese football have been issued in such fields as youth football, women’s football, key city construction, football facility development and field operations, but the effectiveness and scientificity of these policies still require systematic evaluation.

Existing studies on football policies remain relatively scarce, and they focus mainly on such aspects as the effect of anti-hooligan policies on football match ticket sales [4], football club transfer policies [5], the influence of alcohol-banning policies in football stadiums on crime rates [6], the impact of economic regulation policies on the funds of professional football clubs [7], the effect of football policies on football match safety [8], policy misconceptions regarding the football population [9], the characteristics [10] and the evolutionary process of school football policies based on policy text analysis [11], the implementation effects and influencing factors of campus football policies [12], grassroots implementation challenges of campus football policies [13], and football policy innovation [14]. Overall, these studies predominantly emphasize the assessment of individual countries’ football policies, with a prevalence of qualitative research and a relative lack of structured, quantitative analysis. In existing policy analyses, the Policy Modeling Consistency (PMC) index has been widely applied to evaluate college graduate employment policies [15], government service policies [16], and digital economy policies [17]. However, systematic comparative research on Sino-Japanese football policies remains blank. Japan has qualified for the FIFA World Cup for seven consecutive tournaments with stable league development, and its experience is worthy of reference. Hence, through the use of policy instruments and the PMC index model, this study attempts to construct an evaluation framework for Sino-Japanese football policies and systematically conduct a quantitative comparative analysis of policy objectives, policy content, and instrument selections in both countries, so that it can provide reference for the formulation, introduction, implementation, optimization and adjustment of relevant football policies in China.

## 2. Research design

### 2.1. Theoretical framework

This study integrates policy instrument theory and the PMC index model to construct a two-dimensional analytical framework for policy instrument typology and quantitative evaluation of policy quality. Policy instrument theory categorizes policy implementation instruments into five types [18]: authoritative, incentive, capacity-building, systemic reform, and symbolic and hortatory, providing operational coding dimensions for this study. Based on this theory, Table 4 further refines 18 third-level nodes, transforming the content analysis of policy texts from subjective description to structured classification and effectively revealing the preferences and structural imbalances in instrument selection between China and Japan. Meanwhile, the PMC index model, as a quantitative policy evaluation tool, adopts a binary assignment method without preset variable weights [19], enabling objective comparison of policy quality under different governance systems. This model is particularly suitable for analyzing the two significantly different sports governance models: the “state-led” model in China and the “association-led” model in Japan, where the former emphasizes administrative coordination and institutional design, while the latter relies on market-driven mechanisms and industry self-regulation. The comparison between the two cannot be limited to content differences but requires quantifiable comparison under the same evaluation framework. Based on the above theoretical integration, the core contribution of this study is: This study integrates policy instrument theory with quantitative PMC modeling to explain how governance systems shape policy instrument mixes in non-Western sport contexts.

### 2.2. Sources of policy texts

**Fig 1:**
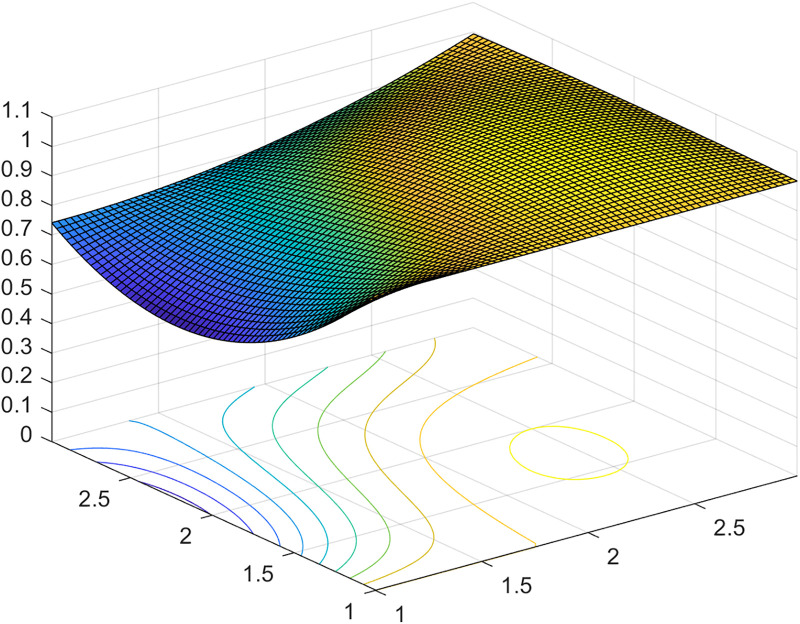
PMC Index Mean Value Radar.

According to collected data from official websites, including the Chinese Government websites, the official portals of the General Administration of Sport of China, the official portal of the Ministry of Education of China, the Japan Football Association (JFA), Japan’s Ministry of Education, Culture, Sports, Science and Technology (MEXT), and Japan’s Ministry of Economy, Trade and Industry (METI), policies with high authority, strong relevance, and regulatory clarity were retrieved with “football” as the keyword [20]. China’s football governance is a governmental act led by multi-department and multi-level authorities. In contrast, Japan’s football governance is dominated by the Football Association, with few policy documents issued by the government. Policy authority and standardization refer to official documents issued by the General Office of the State Council, the General Administration of Sport of China, and the JFA. All regional and provincial policies in China, as well as all prefectural policies in Japan, are excluded from the sample. Relevance means that the policy mainly involves football development, reform, planning and other related content, and the word “football” appears at least 10 times in the policy text. Through retrieval, a total of 18 Chinese policy texts and 13 Japanese policy texts were finally obtained (as shown in Fig 1) as research samples.

### 2.3. Content analysis method

Content analysis constitutes an objective, quantitative method for examining textual materials, which effectively extracts core content from textual sources and, to a certain extent, mitigates the subjectivity and uncertainty inherent in qualitative research [21]. By employing Nvivo 11 to scrutinize Sino-Japanese football policy texts, this study not only identifies high-frequency words within these policies, but also clarifies the application of policy instruments, thus providing a foundation for constructing the PMC evaluation indicators.

### 2.4. PMC index model

The PMC model proposed by Ruiz Estrada is used for multi-dimensional policy quality assessment [19], enabling quantitative comparison and visual presentation. It is one of the internationally advanced and widely adopted policy evaluation instruments [22]. This index model imposes no restrictions on the number or weight of variables and enables the generation of precise surface plots, thereby facilitating an intuitive comparison of the merits and demerits within Sino-Japanese football policies. Constructing the PMC index model involves four steps: (1) variable selection and parameter identification, (2) the establishment of a multi-input-output table, (3) the calculation of the PMC index, and (4) the plotting of the PMC surface. To ensure the comparability of Sino-Japanese football policies, the purposive sampling method was adopted to select 6 policies each from China and Japan related to overall football development planning, children and youth football development, and women’s football development for PMC index analysis.

### 2.5. Governance background of Sino-Japanese football

Football governance in China is a national governmental act led by multi-departmental and multi-level governments. This is mainly because China’s competitive sports adopt the nationwide system. As an important component of the sports cause, football has also followed this development path of government overall planning and administrative force promotion. *The Plan*, promulgated in 2015, was issued by the General Office of the State Council and jointly promoted by multiple ministries and commissions. In addition, football development involves multiple fields such as education and finance, and the government-led form can effectively integrate scattered public resources. This governance model is driven by administrative authority and achieves unified action nationwide through top-down policy deployment.

In contrast, football governance in Japan has formed an industry self-governance model led by the JFA and market-oriented. The JFA was founded in 1921 and possesses independent personnel, financial and policy-making powers. The Japanese government mainly plays a supporting role in football development and does not directly interfere in the internal affairs of the industry. This governance model is oriented by the endogenous needs of the industry and realizes development through market mechanisms and social self-governance.

The analysis of the governance background of Sino-Japanese football provides the institutional foundation for understanding the subsequent differences in the structure of football policy instruments and the divergence in quality characteristics between the two countries.

## 3. Analysis of Sino-Japanese football policy content and policy instruments

### 3.1. Analysis of Sino-Japanese football policy content

#### 3.1.1. Analysis of high-frequency policy words.

This study utilizes the Nvivo 11 software to conduct a quantitative analysis of word frequencies in 31 Sino-Japanese football policy documents. After excluding non-substantive terms such as “football”, “increase”, and “conditions”, we extracted the top 40 high-frequency words in these football policies of the two countries (as shown in Table 1 and Table 2).

**Table 1:** Keyword frequency statistics results of 18 Chinese football policies.

No.	China Keywords	Frequency	No.	China Keywords	Frequency
1	Campus	613	21	General Administration	169
2	Development	572	22	Level	166
3	Sports	392	23	Talent	163
4	Construction	387	24	League	162
5	Youth	384	25	Facilities	158
6	Society	330	26	Women’s Football	157
7	Work	315	27	Education	156
8	Management	275	28	Activities	151
9	Schools	240	29	Departments	150
10	Reform	225	30	Encouragement	147
11	Field	207	31	Cultivation	146
12	System	196	32	Projects	146
13	Support	192	33	Implementation	141
14	Organization	190	34	Exercise	141
15	Mechanism	186	35	Cities	136
16	Competitions	181	36	Training	136
17	Establishment	179	37	Coaching	135
18	National	177	38	Professional	135
19	Improvement	176	39	Events	133
20	Clubs	170	40	Coaches	129

**Table 2:** Keyword frequency statistics results of 13 Japanese football policies.

No.	Japan Keywords	Frequency	No.	Japan Keywords	Frequency
1	JFA	343	21	Promotion	119
2	Activities	289	22	Measures	116
3	Training	251	23	Domain	115
4	World Cup	251	24	Ability	112
5	Matches	243	25	Popularization	110
6	Objectives	228	26	Children	109
7	Players	217	27	Situation	106
8	Plans	203	28	Registration	105
9	Development	202	29	Clubs	104
10	League	192	30	Implementation	103
11	Strengthening	188	31	Personnel	102
12	Coaches	172	32	National Team	101
13	Association	168	33	Foundation	98
14	Environment	153	34	Countermeasures	98
15	Cooperation	147	35	Age Groups	96
16	Organization	142	36	Provision	95
17	Achievement	140	37	Management	95
18	Society	134	38	Formulation	94
19	Sports	124	39	System	88
20	Guidance	120	40	International	85

These high-frequency words represent the primary focal points of these football policies in China and Japan. As shown in Tables 1 and 2, the co-occurring keywords in the 31 football policies mainly include “development”, “society”, “organization”, “competitions”, and “clubs”. This indicates that these policies in both countries place equal emphasis on the social role of football development, the organization of football competitions, and the development of football clubs.

#### 3.1.2. Analysis of policy content based on high-frequency words.

Based on the data shown in Tables 1 and 2, the high-frequency words in the aforementioned football policies include “campus”, “development”, “management”, “schools”, “reform”, and “field” for China; and “JFA”, “activities”, “World Cup”, “players”, “coaches”, and “environment” for Japan. Based on the high-frequency words of China’s football policies in Table 1, several observations can be made. Firstly, China attaches great importance to campus football development and reserve talent cultivation. This unquestionably responds to General Secretary Xi Jinping’s instruction that “it is hoped that a group of outstanding football players will grow up through the development of campus football” [23]. Secondly, China emphasizes football governance encompassing multiple dimensions including competition discipline, event safety, teaching methods, competitive structures, and operational models, which reflects football’s transition into a phase of multi-dimensional and steady development. Thirdly, China has successively promulgated a series of football policies including *The Plan*, *Basic Requirements for National Youth Football Reform Pilot Zones (Trial)*, *China Women’s Football Reform and Development Plan (2022–2035)*, and *Implementation Opinions on the Reform and Development of China’s Youth Football*. This demonstrates that China’s football reform is characterized by its long duration, wide coverage, and in-depth advancement. Fourthly, China prioritizes football facility development, including field construction planning, field facility improvement, and the construction of community football fields, which embodies the Chinese government’s determination to allocate high-quality resources for the promotion of football reform and development.

From the high-frequency words in Japan’s football policies in Table 2, the following observations can be made. First, Japan’s football development is primarily led by JFA. This situation is intrinsically linked to its relatively early establishment, well-structured organizational framework, and sound management system. Second, the reform and development of Japanese football emphasize the cultivation of footballers and coaches. They mainly focus on their growth process and well-being, the naturalization and contributions of overseas footballers and coaches, and the provision of demonstration platforms and learning-exchange opportunities for elite footballers and coaches. Consequently, Japan regards the sense of happiness, expertise cultivation, and capacity demonstration among football professionals as the bedrock and foundation of its football reform and development. Third, Japanese football attaches much importance to environmental optimization to create development opportunities for athletes, which involves training environments, competitive settings, societal contexts, and international exposure. Fourth, JFA places significant emphasis on participating in and organizing World Cup competitions, based on which it defines its World Cup participation goals and strategic pathways.

### 3.2. Analysis of Sino-Japanese football policy instruments

#### 3.2.1. Content coding of policy texts.

This study adopted a purposive sampling method to choose six football policy documents from each of the two countries for coding analysis (as shown in Table 3). During coding, McDonnell’s [18] policy classification framework was applied. Under five secondary nodes of policy instruments, namely authoritative, incentive, capacity-building, system-reforming, and symbolic & exhortative, this study established 18 tertiary nodes, including requirements, regulations, funding, incentives, and institutional construction. Subsequently, adhering to specific coding principles (as shown in Table 4), the policy texts were coded word by word and sentence by sentence.

**Table 3:** 12 Sino-Japanese football policies.

No.	Document Name	Release Date	Issuing Authority
CP_1_	Overall Plan for the Reform and Development of Chinese Football	March 8, 2015	General Office of the State Council
CP_2_	Medium-to Long-term Development Plan for Chinese Football (2016–2050)	April 6, 2016	National Development and Reform Commission and 3 other departments
CP_3_	Guiding Opinions on the Construction of National Key Cities for Football Development	May 19, 2021	General Administration of Sport of China
CP_4_	Reform and Development Plan for Chinese Women’s Football (2022–2035)	October 14, 2022	Ministry of Education and 2 other departments
CP_5_	Implementation Opinions on the Reform and Development of Chinese Youth Football	February 23, 2024	General Administration of Sport of China and 11 other departments
CP_6_	Implementation Opinions on Strengthening and Improving Youth Campus Football in the New Era	February 2, 2024	Ministry of Education and 6 other departments
JP_1_	JFA 2005 Declaration	January 1, 2005	Japan Football Association
JP_2_	JFA Mid-term Plan 2022–2025	January 12, 2022	Japan Football Association
JP_3_	Japan’s Way	July 14, 2022	Japan Football Association
JP_4_	Master Plan for Women’s Football Development	2024	Japan Football Association
JP_5_	JFA Mid-term Plan 2023–2026	March 25, 2023	Japan Football Association
JP_6_	Kids’ Elite Program	April 1, 2009	Japan Football Association

**Table 4:** Categories and definitions of policy instruments.

Secondary Nodes (Instrument Type)	Connotation	Tertiary Nodes (Specific Content)	Manifestations
Authoritative Instruments	Regulate individual and collective behaviors through laws, direct administration, and regulations to achieve policy objectives.	Requirements	Forcibly promote the implementation of football policies to achieve policy effects.
		Regulations	Formulate corresponding laws and regulations.
		Standards	Establish football work norms that policy actors must follow.
		Prohibitions	Prohibit the abuse of related rights.
		Permissions	Authorize relevant rights and formulate corresponding codes of conduct.
Incentive Instruments	Apply positive authorization, rewards, or penalties to policy targets.	Funding	Allocate special funds to support football reform and development.
		Rewards	Commend and reward outstanding individuals and collectives.
		Penalties	Penalize individuals and collectives that fail to meet standards.
Capacity-building Instruments	Provide institutional guarantees, education, training, etc., to individuals or collectives to support policy formulation and activity implementation.	Institutional Development	Formulate and improve working systems to ensure institutional supply.
		Policy Support	Provide additional policy support for football development.
		Platform Construction	Create an environment or conditions for football reform and development.
		Education and Training	Conduct relevant knowledge and skills training.
System-reforming Instruments	Enhance policy execution and legitimacy by reforming the organizational structure of policy targets.	Organization Establishment	Establish relevant responsible departments.
		Organizational Reform	Restructure and adjust existing functional departments.
		Function Definition	Define the responsibilities of relevant functional departments and practitioners.
Symbolic & Exhortative Instruments	Guide target groups to act in accordance with policies through value guidance, assimilation, and other strategies.	Encouragement	Promote football development through motivational means.
		Advocacy	Call on the public to actively participate in football activities.
		Publicity	Promote football development through publicity measures.

#### 3.2.2. Coding reliability verification.

To ensure the validity of policy text coding, two coders conducted independent coding. In case of discrepancies, a third expert arbitrated the disputed items. A total of 47 coding disagreements occurred across 592 reference points (7.9%). Disagreements were most frequent in the tertiary nodes ‘Platform Construction’ (n = 12) and ‘Functional Delineation’ (n = 9). The third expert was a professor of sport policies with 10 years of experience. All unresolved items were discussed in two consensus meetings. Coding consistency was also verified [24]. After coding, one Chinese football policy document and one Japanese football policy document were randomly selected, and the Kappa coefficient in Nvivo was used to test the consistency of coding results between the two coders, ensuring the reliability of coding at all nodes. Generally, a Kappa value exceeding 0.80 signals the perfect consistency. In other words, a Kappa value above 0.80 conforms to the criteria of reliability verification [25]. According to verification results, the Kappa values for all nodes in the 12 selected policies exceeded 0.87, which satisfied the criteria of reliability verification.

#### 3.2.3. Commonality analysis of Sino-Japanese football policies based on policy instruments.

While the 12 Sino-Japanese football policies exhibit their respective distinct features in terms of the types and structures of policy instruments, they share extensive commonalities in core content. These commonalities undoubtedly represent the shared visions, aspirations, and strategic planning for football development in both countries. In accordance to the aforementioned coding method, 333 Chinese reference points and 259 Japanese reference points were accessed. Subsequently, the coverage rates for secondary and tertiary nodes were calculated based on node quantities (as detailed in Table 5).

**Table 5:** Analysis results of 12 Sino-Japanese football policies based on policy instruments.

Secondary Nodes	Tertiary Nodes	Chinese Material Sources	Chinese Reference Points	Chinese Coverage Rates	Japanese Material Sources	Japanese Reference Points	Japanese Coverage Rates
Authoritative Instruments	Requirements	6	61	69%	5	34	97%
	Regulations	6	12	13%	1	1	3%
	Standards	6	7	8%	0	0	0%
	Prohibitions	1	1	1%	0	0	0%
	Permissions	5	8	9%	0	0	0%
	Subtotal		89	25%		35	14%
Incentive Instruments	Funding	6	17	63%	1	5	100%
	Rewards	3	3	11%	0	0	0%
	Penalties	4	7	26%	0	0	0%
	Subtotal		27	10%		5	2%
Capacity-building Instruments	Institutional Development	6	58	35%	4	29	15%
	Policy Support	6	15	9%	1	3	2%
	Platform Construction	6	56	34%	6	93	49%
	Education and Training	6	38	23%	6	64	34%
	Subtotal		167	50%		189	73%
System-reforming Instruments	Organization Establishment	3	7	58%	0	0	0%
	Organizational Reform	2	3	25%	3	5	83%
	Functional Delineation	2	2	17%	1	1	17%
	Subtotal		12	4%		6	2%
Symbolic & Exhortative Instruments	Encouragement	6	25	66%	2	4	17%
	Advocacy	3	3	8%	3	12	50%
	Publicity	6	10	26%	3	8	33%
	Subtotal		38	11%		24	9%

According to the statistics of the secondary nodes in Table 5, the coverage rates of reference points for “capacity-building instruments” in these selected Sino-Japanese football policies stand at 50% and 73% respectively, while those for “authoritative instruments” are respectively 25% and 14%. This indicates that policymakers in both countries take capacity-building and authoritative instruments as paramount priorities. Consequently, these instruments receive considerable attention and resource allocation in policy formulation. Among these, capacity-building instruments primarily contain football talent development, competition organization enhancement, on-field performance improvement, coaching cultivation, and the expansion of female participation. Authoritative instruments cover football development initiatives and team-building frameworks. In terms of tertiary nodes, the 12 football policies in both China and Japan generally focus on policy requirements, with coverage rates of 69% and 97% respectively, and funding support, with coverage rates of 63% and 100% respectively. This practice serves two purposes: (1) Taking policy requirements as the leverage to provide directional guidance and institutional guarantee for football development. (2) Relying on financial support as the foundation to offer funding guarantee and operational support for football development.

#### 3.2.4. Difference analysis of 12 Sino-Japanese football policies based on policy instruments.

As a means of translating policy intentions into policy actions, policy instruments are irreversible institutional arrangements and also an inexhaustible force ensuring football development. Their configuration determines the direction and quality of football development. As presented in Table 5, there exist significant differences in data sources and reference points across different secondary and tertiary nodes. As stated earlier, it is precisely the different governance contexts of Sino-Japanese football that lead to the differences in the use of football policy instruments between the two countries. The exploration of these concrete differences requires a further analysis of the node-coded content.

Regarding the authoritative instrument node, the six Chinese football policies primarily concern four tertiary nodes: requirements, regulations, standards, and permissions. It follows that these Chinese football policies represent the national will and provide essential necessary institutional guarantees for football development. In contrast, among the six Japanese football policies, only five documents refer to requirements and one involves regulations. This reveals a markedly uneven structure in policy instruments of Japanese football, which is intrinsically linked to the reality that JFA determines the direction of Japanese football development. After all, due to the lack of its administrative status, JFA exposes its obvious shortcomings and disadvantages in advancing regulation formulation, standard establishment, permissions and prohibitions.

With regard to the incentive instrument node, the six Chinese football policies involve such issues as football funding support and the expansion of financial channels. They not only demand government financial guarantees through fiscal expenditure and tax incentives but also motivate social capital infusion. Concurrently, these policies impose penalties on irregular competition activities, violations of laws and regulations, unauthorized alterations to land-using, and key cities with unqualified football development evaluations. While the six Japanese football policies do not explicitly touch upon government funding support, *JFA Mid-term Plan 2023–2026* refers to funding using. In actual fact, these Japanese football policies put much stress on fund guarantee through financial support, including commercial partnerships within the football associations, revenues from events, and social donations.

Regarding the capacity-building instrument node, these selected Chinese football policies pay much attention to institutional development and platform construction. Institutional development primarily encompasses three aspects: mechanisms, systems, and institutions. Among them, mechanisms chiefly include resource sharing, talent cultivation, funding allocation, and club supervision. Systems contain training, competition, evaluation, and management. Institutions involve electronic talent profiles, club management, league coordination, and inter-ministerial joint meetings. Platform construction covers such dimensions as infrastructure construction, youth echelon development, club development, social support, and exchange promotion. The selected Japanese football policies regard platform construction as the key point of football development and focus on the reinforcement of football‑related education and training. Platform construction mainly involves competitions and cooperation. On one hand, these policies create many diversified opportunities for football players, stage many events such as professional leagues, university leagues, East‑West leagues, women’s professional leagues, the J. League (Japan Professional Football League), and national leagues, and provide systematic analyses of match data and high-quality match environment. On the other hand, these policies employ the cooperative method to enhance football skills, cultivate football players, conduct popularization activities, achieve high added value, and expand fan bases. Japanese football training gives priority to the quantity, quality, system, planning, methods, process, and model of football talent cultivation. Firstly, it lays emphasis on cultivating players’ technical skills and decision-making abilities, and proposes to utilize varied match tempos and pressure scenarios to enhance their adaptability, physical confrontation ability, and mental resilience. Through diverse competition opportunities and professional team guidance, it works to improve players’ individual tactics, teamwork skills, and over-round development levels. Secondly, it focuses on training outstanding coaches and improving their ability of individualized teaching, so that they can exploit players’ potentials and thereby heighten the quality of football talent cultivation. In the process of learning and training, football players can experience joy and growth, while developing their independent thinking, self-management, and self-directed development.

In terms of the systemic-reform instrument node, the selected Chinese football policies place particular emphasis on organizational establishment, which gives priority to the establishment of non‑governmental organizations, with the establishment of governmental organizations supplementary. The established non-governmental organizations primarily include youth training institutions with industry-leading and exemplary social football brands, amateur football clubs and regional non-professional football leagues, professional league management bodies established by local and industry football associations, and dedicated women’s football management teams. On the governmental side, high priority should be given to establishing leadership teams for key cities’ football development and their vital functional departments. JFA’s systemic-reforming instruments primarily address organizational reform and functional delineation, with a notable absence in organizational establishment. Its organizational reform is aimed at building powerful, trustworthy institutions and organizations which can confront social crises to construct new benefit cycle models. Furthermore, functional delineation stresses the strengthening of coaches’ responsibilities: (1) They should be learning guides, interest motivators, and personal development shapers for footballers. (2) They should standardize and optimize footballers’ development pathways.

Regarding the symbolic & exhortative instrument node, the six Chinese football policies adopt an encouragement-based approach. Firstly, these policies encourage localities to actively carry out campus football programs. Secondly, they encourage social forces to participate in football reform and development. Thirdly, they prioritize the cultivation of reserve talents and the optimization and integration of resources. All of them can serve to reinforce human resource support and guarantee for football, refine football operation mechanisms, and enhance the efficiency and quality of football reform and development. By contrast, the six Japanese football policies attach much importance to advocacy. Through policy advocacy, these policies seek to strengthen football developmental ideas and cultural functions, provide the spiritual core and value foundation for the sport, and promote the diverse and integrated development of Japanese football. Accordingly, they can attract more children and youth to participate in this sport, increase football participation population, and facilitate the sustainable development of football.

## 4. Quantitative analysis of 12 Sino-Japanese football policies based on the PMC index model

### 4.1. Variable selection and parameter identification

As a crucial step in building the PMC index model, variable selection can, to a certain extent, influence the scientific validity and rationality of policy evaluation results. Therefore, variable selection should strive for comprehensiveness wherever possible [26]. In building the PMC index model, this study refers to policy quantification indicators proposed by scholars such as Ruiz Estrada [19] and Mei Shuwen [27], and incorporates high-frequency words to set variable indicators. The selection of variable indicators mainly considers whether to objectively reflect policy content and whether to reveal the trends and features of football development. Through two rounds of screening with the Delphi method, ten primary variable indicators and forty-nine secondary variable indicators were ultimately determined (see Table 6). Based on the selected twelve Sino-Japanese football policy documents, Secondary variables were assigned using the internationally adopted binary method with equal weights: if a football policy text involves information on a secondary variable, the variable is assigned a value of 1; otherwise, it is assigned a value of 0 [15,17].

**Table 6:** Evaluation indicator system and sources of 12 Sino-Japanese football policies.

Primary Variables	Secondary Variables	Parameter Setting	Primary Variable Sources
X_1_ Policy Types	X_1-1_ Prediction	1 for yes, 0 for no: Whether it predicts future prospects	Revised based on Ruiz Estrada’s article
	X_1-2_ Suggestion	1 for yes, 0 for no: Whether it provides development strategies	
	X_1-3_ Supervision	1 for yes, 0 for no: Whether it involves supervision and management	
	X_1-4_ Description	1 for yes, 0 for no: Whether it describes development status	
	X_1-5_ Guidance	1 for yes, 0 for no: Whether it provides goal-oriented guidance	
X_2_ Policy Timeframe	X_2-1_ Long-term	1 for yes, 0 for no: Whether it involves long-term planning (>5 years)	Revised based on Mei Shuwen’s article
	X_2-2_ Medium-term	1 for yes, 0 for no: Whether it involves medium-term planning (≥3 and ≤5 years)	
	X_2-3_ Short-term	1 for yes, 0 for no: Whether it involves short-term planning (≥1 and <3 years)	
	X_2-4_ Ultra-short-term	1 for yes, 0 for no: Whether it involves ultra-short-term planning (≤1 year)	
X_3_ Policy Perspective	X_3-1_ Macro	1 for yes, 0 for no: Whether it involves macro aspects	Revised based on Zhai Yun’s article [28]
	X_3-2_ Meso	1 for yes, 0 for no: Whether it involves meso aspects	
	X_3-3_ Micro	1 for yes, 0 for no: Whether it involves micro aspects	
X_4_ Policy Objectives	X_4-1_ Expand Football Population	1 for yes, 0 for no: Whether it involves expanding football population	Revised based on policy text mining
	X_4-2_ Improve Competitive Levels	1 for yes, 0 for no: Whether it involves improving competitive levels	
	X_4-3_ Foster Cultural Atmosphere	1 for yes, 0 for no: Whether it involves fostering cultural atmosphere	
	X_4-4_ Participate in World Cup	1 for yes, 0 for no: Whether it involves participating in World Cup	
	X_4-5_ Participate in Olympics	1 for yes, 0 for no: Whether it involves participating in Olympics	
X_5_ Policy Content	X_5-1_ Social Participation	1 for yes, 0 for no: Whether it involves social participation	Revised based on policy text mining
	X_5-2_ School Promotion	1 for yes, 0 for no: Whether it involves school promotion	
	X_5-3_ Talent Cultivation	1 for yes, 0 for no: Whether it involves talent cultivation	
	X_5-4_ Facility Construction	1 for yes, 0 for no: Whether it involves facility construction	
	X_5-5_ Competition Organization	1 for yes, 0 for no: Whether it involves competition organization	
	X_5-6_ Association Operation	1 for yes, 0 for no: Whether it involves association operation	
	X_5-7_ Club Operation	1 for yes, 0 for no: Whether it involves club operation	
	X_5-8_ Athletes’ Rights and Interests	1 for yes, 0 for no: Whether it involves athletes’ rights and interests	
	X_5-9_ Increasing Registered Players	1 for yes, 0 for no: Whether it involves increasing registered players	
	X_5-10_ International Exchange	1 for yes, 0 for no: Whether it involves international exchange	
	X_5-11_ Family Support	1 for yes, 0 for no: Whether it involves family support	
	X_5-12_ Coach Training	1 for yes, 0 for no: Whether it involves coach training	
	X_5-13_ Sports Spirit	1 for yes, 0 for no: Whether it involves sports spirit	
X_6_ Policy Instruments	X_6-1_ Authoritative	1 for yes, 0 for no: Whether it involves authoritative instruments	Revised based on Mcdonnell L’s article
	X_6-2_ Incentive	1 for yes, 0 for no: Whether it involves incentive instruments	
	X_6-3_ Capacity-building	1 for yes, 0 for no: Whether it involves capacity-building instruments	
	X_6-4_ System-reforming	1 for yes, 0 for no: Whether it involves system-reforming instruments	
	X_6-5_ Symbolic & Exhortative	1 for yes, 0 for no: Whether it involves symbolic & exhortative instruments	
X_7_ Policy Evaluation	X_7-1_ Clear Objectives	1 for yes, 0 for no: Whether objectives are clear	Revised based on Zhang Yong’an’s article [29]
	X_7-2_ Sufficient Basis	1 for yes, 0 for no: Whether basis is sufficient	
	X_7-3_ Detailed Content	1 for yes, 0 for no: Whether content is detailed	
	X_7-4_ Scientific Schemes	1 for yes, 0 for no: Whether schemes are scientific	
X_8_ Policy Clarity	X_8-1_ Clear Timeframe	1 for yes, 0 for no: Whether completion time is specified	Revised based on Tan Hao’s article [30]
	X_8-2_ Clear Indicators	1 for yes, 0 for no: Whether specific indicators are defined	
	X_8-3_ Clear Responsible Entities	1 for yes, 0 for no: Whether responsible entities are specified	
	X_8-4_ Clear Resource Guarantee	1 for yes, 0 for no: Whether resource allocation is specified	
	X_8-5_ Clear Reward/Penalty Measures	1 for yes, 0 for no: Whether reward/penalty measures are defined	
X_9_ Policy Targets	X_9-1_ Government Departments	1 for yes, 0 for no: Whether targeted at government departments	Revised based on Ma Wenyou’s article [31]
	X_9-2_ General Public	1 for yes, 0 for no: Whether targeted at general public	
	X_9-3_ Schools	1 for yes, 0 for no: Whether targeted at schools	
	X_9-4_ Associations	1 for yes, 0 for no: Whether targeted at associations	
	X_9-5_ Clubs	1 for yes, 0 for no: Whether targeted at clubs	
	X_9-6_ National Teams	1 for yes, 0 for no: Whether targeted at national teams	
X_10_ Policy Publicity	(Not specified)	(Not specified)	Revised based on Ruiz Estrada’s article [32]

### 4.2. Construction of the multi-input-output table

A multi-input-output table can store a large quantity of data and assess policy effectiveness from multiple dimensions. Variables are mutually independent, with no sequential order. In accordance with the evaluation index system for football policy effectiveness in China and Japan, a multi-input-output table is constructed (as shown in Table 7).

**Table 7:** Multi-input-output table of 12 Sino-Japanese football policies.

Primary Variables	Secondary Variables	CP_1_	CP_2_	CP_3_	CP_4_	CP_5_	CP_6_	JP_1_	JP_2_	JP_3_	JP_4_	JP_5_	JP_6_
X_1_	X_1-1_	1	1	1	1	1	1	1	1	1	1	1	1
	X_1-2_	0	0	1	0	1	1	0	0	1	0	0	1
	X_1-3_	1	1	1	1	1	1	0	0	0	0	0	0
	X_1-4_	1	1	1	1	1	1	1	1	1	0	1	1
	X_1-5_	1	1	1	1	1	1	1	1	1	1	1	1
X_2_	X_2-1_	1	1	1	1	1	1	1	1	1	1	1	1
	X_2-2_	1	1	1	1	1	1	1	1	0	0	1	0
	X_2-3_	1	1	1	1	1	1	0	0	0	1	1	0
	X_2-4_	0	1	1	1	0	0	0	0	1	1	1	0
X_3_	X_3-1_	1	1	1	1	1	1	0	1	1	1	1	1
	X_3-2_	1	1	1	1	1	1	1	1	1	1	1	1
	X_3-3_	1	1	1	1	1	1	1	1	1	1	1	1
X_4_	X_4-1_	1	1	1	1	1	1	1	1	1	0	1	1
	X_4-2_	1	1	1	1	1	1	1	1	1	1	1	1
	X_4-3_	1	1	1	1	1	1	1	1	1	0	0	0
	X_4-4_	0	0	0	0	0	0	1	1	1	1	1	0
	X_4-5_	0	0	0	1	0	0	0	1	0	0	1	0
X_5_	X_5-1_	1	1	1	1	1	0	0	0	1	0	1	0
	X_5-2_	1	1	1	1	1	1	0	1	0	1	0	1
	X_5-3_	1	1	1	1	1	1	1	1	1	1	1	1
	X_5-4_	1	1	1	1	1	1	0	0	0	0	0	0
	X_5-5_	1	1	1	1	1	1	1	1	1	1	1	1
	X_5-6_	1	1	1	1	1	1	0	1	0	1	1	1
	X_5-7_	1	1	1	1	1	1	1	1	1	1	1	1
	X_5-8_	1	1	1	1	1	1	0	0	0	0	0	0
	X_5-9_	0	1	1	0	0	0	0	1	0	1	1	0
	X_5-10_	1	1	1	1	1	1	0	1	0	0	1	0
	X_5-11_	1	1	1	0	1	1	1	1	1	0	0	1
	X_5-12_	1	1	1	1	1	1	1	1	1	1	0	1
	X_5-13_	1	1	1	1	1	1	1	1	1	0	1	1
X_6_	X_6-1_	1	1	1	1	1	1	1	1	1	1	0	0
	X_6-2_	1	1	1	0	1	1	0	0	0	0	1	1
	X_6-3_	1	1	1	1	1	1	1	1	1	1	1	1
	X_6-4_	1	1	1	1	1	1	0	1	1	1	1	0
	X_6-5_	1	1	1	1	1	1	1	1	1	1	1	0
X_7_	X_7-1_	1	1	1	1	1	0	1	1	1	1	1	1
	X_7-2_	0	1	1	1	1	1	1	1	1	0	1	1
	X_7-3_	1	1	1	0	1	1	0	1	1	0	1	0
	X_7-4_	1	1	0	1	1	1	0	1	1	1	1	0
X_8_	X_8-1_	1	1	1	1	1	1	1	1	1	1	1	0
	X_8-2_	1	1	1	1	1	0	1	1	1	1	1	0
	X_8-3_	0	0	0	1	1	1	0	1	0	0	0	0
	X_8-4_	0	0	1	0	1	0	0	0	0	0	0	0
	X_8-5_	0	0	1	0	0	0	0	0	0	0	0	0
X_9_	X_9-1_	0	1	1	1	1	1	0	0	0	0	0	0
	X_9-2_	0	1	1	0	0	0	0	0	0	0	0	0
	X_9-3_	1	1	1	1	1	1	0	1	0	1	0	1
	X_9-4_	1	1	1	1	1	1	0	1	1	1	1	1
	X_9-5_	1	1	1	1	1	1	1	1	1	1	1	1
	X_9-6_	1	0	1	1	1	0	0	0	1	1	1	0
X10	None	1	1	1	1	1	1	1	1	1	1	1	1

### 4.3. Calculation of the PMC index

The calculation of the PMC Index is comprised of three steps: first, according to Formula (1) and Formula (2), secondary variables are assigned values. Second, according to Formula (3), the values of each primary variable are calculated. Finally, according to Formula (4), the values of primary variables are summed to produce the PMC indices of Sino-Japanese football policies. The indices are then graded according to the policy grading classification criteria [33] (as shown in Table 8).

**Table 8:** Grading Classification of 12 Sino-Japanese Football Policies.

PMC Index	0 ~ 2.99	3 ~ 4.99	5 ~ 6.99	7 ~ 8.99	9 ~ 10
Grades	Poor	Acceptable	Good	Excellent	Perfect


$$\begin{array}{c} X\;N\left[0,1\right] \end{array}$$ (1)

$$\begin{array}{c} X=\left\{XR:\left[0,1\right]\right\} \end{array}$$ (2)

$$\begin{array}{c} X_{\text{t}}\left(\sum_{j=1}^{\text{n}}\frac{X_{tj}}{T\left(X_{tj}\right)}\right),t=1,2,3,4,\cdot \cdot \cdot ,\infty \end{array}$$ (3)

$$\begin{array}{c} \begin{array}{c} PMC=[X_{\text{1}}\left(\sum_{t=1}^{\text{5}}\frac{X_{1t}}{\text{5}}\right)+X_{\text{2}}\left(\sum_{j=1}^{\text{4}}\frac{X_{2j}}{\text{4}}\right)+X_{\text{3}}\left(\sum_{k=1}^{\text{3}}\frac{X_{3k}}{\text{3}}\right)+X_{\text{4}}\left(\sum_{l=1}^{\text{5}}\frac{X_{4l}}{\text{5}}\right)+X_{\text{5}}\left(\sum_{m=1}^{13}\frac{X_{5m}}{13}\right)+\\ \;X_{\text{6}}\left(\sum_{n=1}^{\text{5}}\frac{X_{6n}}{\text{5}}\right)+X_{\text{7}}\left(\sum_{o=1}^{\text{4}}\frac{X_{7o}}{\text{4}}\right)+X_{\text{8}}\left(\sum_{p=1}^{\text{5}}\frac{X_{8p}}{\text{5}}\right)+X_{\text{9}}\left(\sum_{q=1}^{\text{6}}\frac{X_{9q}}{\text{6}}\right)+X_{10}] \end{array} \end{array}$$ (4)

$$\begin{array}{c} PMC=\left[\begin{array}{ccc} \begin{array}{c} X_{\text{1}}\\ X_{\text{4}}\\ X_{\text{7}} \end{array} & \begin{array}{c} X_{\text{2}}\\ X_{\text{5}}\\ X_{\text{8}} \end{array} & \begin{array}{c} X_{\text{3}}\\ X_{\text{6}}\\ X_{\text{9}} \end{array} \end{array}\right] \end{array}$$ (5)

### 4.4. Plotting of the PMC surface

**Fig 2:**
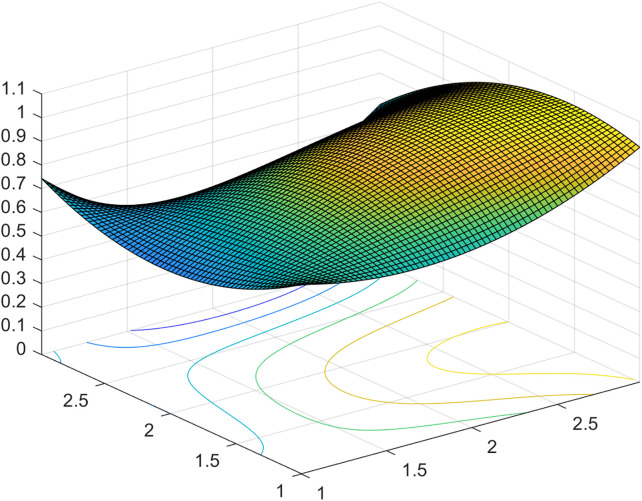
CP_1_ surface plot.

**Fig 3:**
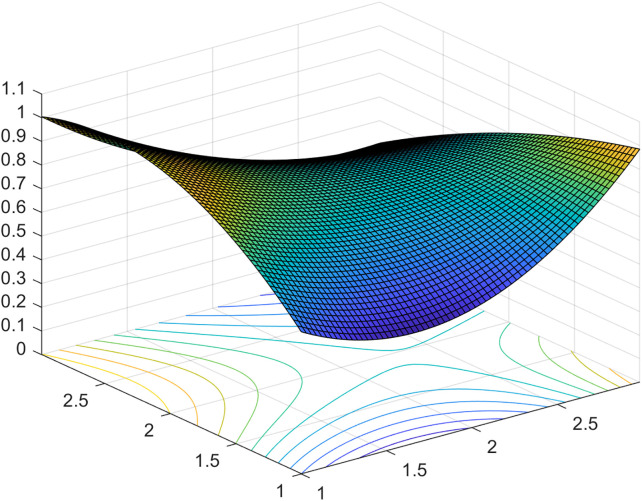
JP_2_ surface plot.

**Fig 4:**
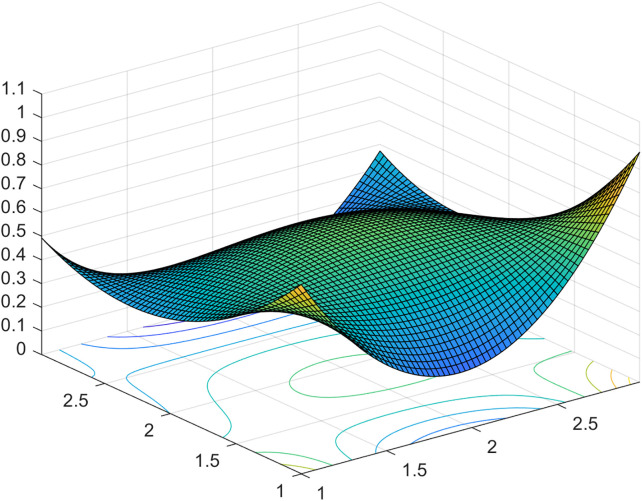
JP_6_ surface plot.

**Fig 5:**
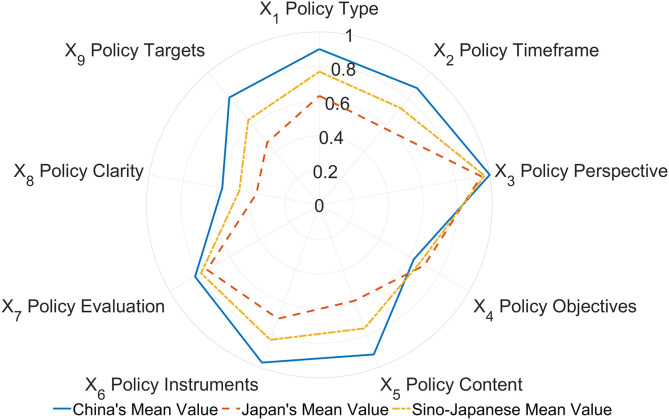
PMC Index Mean Value Radar Chart: Sino-Japanese.

The PMC surface plot can matrix the first-level variable values of each policy, and the advantages and disadvantages of the policy can be directly observed through the surface plot. Given the symmetry of the surface, the variable values for X_10_ are excluded. A 3 × 3 matrix is constructed via Formula (5), and the surface plot is produced using MATLAB. The deeper the concavity of the surface, the darker the cool tones of the surface and the base contour lines, which indicates a lower PMC index and a higher concavity index for the policy. Conversely, the stronger the warm tones of the surface and the base contour lines, the higher the PMC index and the lower the concavity index. Owing to space constraints, this article displays only the surface plots with the highest and lowest PMC indices for Sino-Japanese football policies, namely CP_3_ (Fig 2), CP_1_ (Fig 3), JP_2_ (Fig 4), and JP_6_ (Fig 5).

### 4.5. Quantitative evaluation of 12 Sino-Japanese football policies

#### 4.5.1. Overall evaluation of 12 Sino-Japanese football policies.

**Fig 6:**
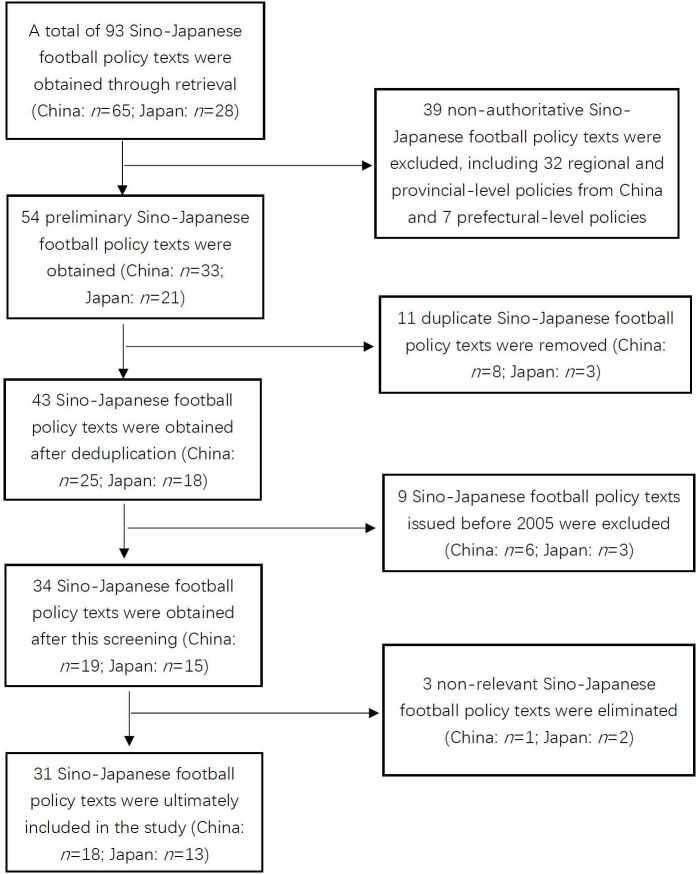
Sino-Japanese Football Policy Screening Flowchart.

Based on the PMC index calculation method and the policy grading classification criteria, the PMC indices and corresponding grades for 12 Sino-Japanese football policies were produced (as shown in Table 9). Among these policies, only CP_3_ achieved the grade of “Perfect”. Eight policies, including CP_1_, CP_2_, JP_1_, and JP_3_, were classified as “excellent”. JP_1_ and JP_4_ were rated as “good”; and JP_6_ was assessed as “acceptable”. The mean value of the overall PMC indices stood at 7.59. A radar chart of the PMC index mean value for 12 Sino-Japanese football policies was generated using MATLAB (see Fig 6), which visually illustrated the overall merits and demerits of the two countries’ 12 football policies. Here in Fig 5, there is an exception in the mean values for the primary variables X_4_ (Policy Objectives), where China’s mean value was slightly lower than Japan’s. Except that, the mean values for all other primary variables exceeded those of Japan. For China’s six policies, the mean values were relatively higher ordinally in X_3_ (Policy Perspective), X_6_ (Policy Instruments), X_5_ (Policy Content), and X_1_ (Policy Types), while relatively lower in X_8_ (Policy Clarity), X_4_ (Policy Objectives), X_9_ (Policy Targets), and X_7_ (Policy Evaluation). For Japan’s six policies, the mean values ranked higher in X_3_ (Policy Perspective), X_7_ (Policy Evaluation), X_6_ (Policy Instruments), and X_1_ (Policy Types), with relatively lower mean values in X_8_ (Policy Clarity), X_9_ (Policy Targets), X_2_ (Policy Timeframe), and X_5_ (Policy Content). As indicated in mean values of primary variables, both China and Japan obtained low values in policy clarity. In China’s case, inadequate policy clarity was mainly ascribed to the top-down design and hierarchical implementation of its sports policies, which tended to result in ambiguous definitions of specific details. For Japan, the absence of policy clarity in these football policies was attributed to its market-led policy framework, so that, in formulating the policies, it cannot better balance the demands of multiple entities, including football associations, professional league bodies, clubs, and local governments, which, in turn, results in ambiguous policy statements. This indicates that, as directional guides and driving forces for promoting football development, all these Sino-Japanese football policies demonstrate strong scientific validity and rationality, and yet still leave certain room for further optimization.

**Table 9:** PMC indices and grade evaluation of 12 Sino-Japanese football policies.

Primary Variables	CP_1_	CP_2_	CP_3_	CP_4_	CP_5_	CP_6_	JP_1_	JP_2_	JP_3_	JP_4_	JP_5_	JP_6_	Mean values of primary variables
X_1_	0.8	0.8	1	0.8	1	1	0.6	0.6	0.8	0.4	0.6	0.8	0.77
X_2_	0.75	1	1	1	0.75	0.75	0.5	0.5	0.5	0.75	1	0.25	0.73
X_3_	1	1	1	1	1	1	0.67	1	1	1	1	1	0.97
X_4_	0.6	0.6	0.6	0.8	0.6	0.6	0.8	1	0.8	0.4	0.8	0.4	0.63
X_5_	0.92	1	1	0.85	0.92	0.85	0.46	0.77	0.54	0.54	0.62	0.62	0.76
X_6_	1	1	1	0.8	1	1	0.6	0.8	0.8	0.8	0.8	0.4	0.83
X_7_	0.75	1	0.75	0.75	1	0.75	0.5	1	1	0.5	1	0.5	0.79
X_8_	0.4	0.4	0.8	0.6	0.8	0.4	0.4	0.6	0.4	0.4	0.4	0	0.47
X_9_	0.67	0.83	1	0.83	0.83	0.67	0.17	0.5	0.5	0.67	0.5	0.5	0.64
X_10_	1	1	1	1	1	1	1	1	1	1	1	1	1
PMC Index	7.89	8.63	9.15	8.43	8.9	8.02	5.7	7.77	7.34	6.46	7.72	5.47	7.59
Concavity Index	2.11	1.37	0.85	1.57	1.1	1.98	4.3	2.23	2.66	3.54	2.28	4.53	2.41
Rank	6	3	1	4	2	5	11	7	9	10	8	12	
Grade	Excellent	Excellent	Perfect	Excellent	Excellent	Excellent	Good	Excellent	Excellent	Good	Excellent	Acceptable	

Based on the further exploration of the inherent mechanisms and operational frameworks of Sino-Japanese football policy formulation, a comparative analysis of the summed values of their secondary indicators was conducted by incorporating the multi-input-output table. The results show that, through the promulgations of their medium-and long-term football development plans, both China and Japan clarify their policy objectives, steer their developmental directions, enhance the operation and management of football clubs, and prioritize the cultivation of football talents and the advancement of competitive performance. Nevertheless, the selected Chinese football policies suffer from deficiencies in such aspects as policy recommendations, World Cup participation objectives, registered player increase, policy actors’ responsibilities, resource guarantees, and reward/penalty measures. In contrast, the selected Japanese football policies have defects in policy supervision, short-term planning, social participation, the protection of athletes’ rights and interests, international exchanges, and resource guarantees.

The formulation of football policies in any country is inextricably linked to its then national conditions and developmental context. Therefore, these football policies in both China and Japan are formulated under their respective national situations, with their own distinct features. Compared to Japan, China’s six football policies display higher summed values in such aspects as supervision, medium-and short-term planning, cultural atmosphere, social participation, facility construction, athletes’ rights and interests, and incentive-based policy instruments. Japan Men’s National Football Team has qualified for seven consecutive World Cups since their debut in the 1998 tournament in France, and progressed beyond the group stage on four occasions—in 2002, 2010, 2018, and 2022. This is also why 5 out of the 6 football policies analyzed using the PMC index model mention World Cup participation goals. This not only demonstrates Japan’s competitive performance but also attests to the continuity and stability of its football policy objectives. For instance, in the policy document *JFA Mid-term Plan 2022–2025*, Japan set its medium-and long-term objectives for participating in the men’s and women’s World Cups, the FIFA Futsal World Cup, and the FIFA Beach Soccer World Cup. Furthermore, Japan’s six football policies attach considerable importance to the expansion of football registered population. For example, JFA has reversed the decline in registered players by pushing for reforms in the registration system and prioritizing women and youth as key target groups, which ensured sustained growth in Japan’s football population.

#### 4.5.2. Evaluation of 12 Sino-Japanese representative football policies.

By comparing the content of Table 7, Table 9 and Figs 2–6 this study conducts a comparative analysis of the 12 representative football policies in China and Japan. It is evident that China’s CP_3_, *Guiding Opinions on the Construction of National Key Cities for Football Development*, is rated as “perfect”, and CP_1_, *The Plan*, as “excellent”. Among Japan’s policies, JP_2_, *JFA Mid-term Plan 2022–2025*, is rated as “excellent”, and CP_6_, *Kids’ Elite Program*, as “acceptable”.

CP_3_ employs the development of key football cities as a breakthrough point to explore a football development model suitable for China’s national conditions. This policy provides replicable and transferable successful experience for the advancement of Chinese football cause, and plays a crucial role in driving broader progress through targeted initiatives. As illustrated by Table 9 and Fig 2, CP_3_ yields the highest PMC index among the 12 Sino-Japanese football policies. While the variable values for X_4_ (Policy Objectives), X_7_ (Policy Evaluation), and X_8_ (Policy Clarity) are slightly lower, the variable values for X_1_ (Policy Types), X_2_ (Policy Timeframe), X_3_ (Policy Perspective), X_5_ (Policy Content), X_6_ (Policy Instruments), and X_9_ (Policy Targets) all stand as 1. It can be seen that CP_3_ comprises multiple domains including professional football, youth football, and grassroots football, and provides relatively systematic and comprehensive planning across such aspects as management systems, competition structures, talent development, football culture cultivation, and facility construction, all of which has charted a course and offered pathways for establishing key football cities.

CP_1_ proposes the principle of separating government administration from social organizations. This practice works as a guiding document for Chinese football development but also marks a shift of China’s football management system from traditional models towards modernization, professionalization and marketisation. It establishes long-term objectives, charts a macro-level direction and provides institutional guarantee for the sustainable development of football. However, due to its relatively early introduction, this policy has several deficiencies, namely, unclear responsible entities regarding who is responsible for reform, governance, construction and supervision; unexplicit content and methods for resource integration and reward/penalty measures; unfocused policy objectives concerning participation in international competitions such as the FIFA World Cup; and incomplete coverage of policy targets. These shortcomings result in limited transferable policy experience and make CP_1_ have the lowest PMC index among the selected Chinese football policies. It can be seen from Fig 3 that the policy exhibits a relatively significant depression in X_8_ (Policy Clarity). Its optimization path can be expressed as: X_8_-X_4_-X_9_-X_2_-X_7_-X_8_-X_5_.

JP_2_ is closely associated with such elements as football popularization and promotion, football talent cultivation, organizational management optimization, and commercial value exploration, all of which allow it to obtain the highest PMC index in Japan’s football policies. According to Fig 4, JP2 shows certain depressions in X_2_ (Policy Timeframe) and X_9_ (Policy Targets), indicating that this policy not only lacks short-term and ultra-short-term plans but also has an unclear division of responsibilities between government departments and clubs. Within this policy, the variable values are all 1 in such aspects as X_3_ (Policy Perspective), X_4_ (Policy Objectives) and X_7_ (Policy Evaluation). On the whole, this policy adheres to the sustainable development concepts to advance the football popularization and enhance the national health level. From the social aspect, it attaches much importance to the integrative development of football and society, and also places emphasis on the close cooperation of football associations and sponsors and cooperative partners, so that more commercial value can be made for the football development. Additionally, it puts stress on utilizing such measures as the organizational management of football associations, the construction of new marketing models, and digital and intelligent empowerment to improve football performance and influence. In terms of football families, according to JP_2_, the number of football families is projected to surpass 10 million by 2050. The connection with football families should be strengthened to create favorable family football atmosphere. From the personal aspect, individuals’ active participation in football should be strongly promoted. However, the variable values are relatively lower in X_2_ (Policy Timeframe) and X_9_ (Policy Targets) of this policy, which not only lacks short-term and ultra-short-term plans, but also reveals the unclear responsibility distinction between government departments and clubs.

JP_6_ adopts “the training model that integrates coach training, youth nurturing and representative team strengthening plus grassroots promotion” to provide children and youth with a favorable football ecological environment and targeted guidance plans, and thus ensures their positive participation in and strong penchant for the football sports. There is no doubt that this training model is not only conducive to finding talented and potential children and youth, but also instrumental in establishing a sustainable training system of football talents and also increasing the football population base. Specifically, this policy focuses on illustrating football development concepts with concrete cases. This practice can accelerate the effective unification of football development ideas and concepts, and promote such groups as football practitioners and parents to understand, support and participate in this sport. It can be seen from Fig 5 that this policy has obvious deficiencies in X_2_ (Policy Timeframe), X_4_ (Policy Objectives), X_6_ (Policy Instruments) and X_8_ (Policy Clarity), because it ignores specific implementation plans and details.

## 5. Conclusions and suggestions

Football policies are significant plans and measures that promote and instruct football development. They also work as the fundamental guideline and the crucial driving force of its development. This paper employs the content analysis method and the PMC index model to evaluate Sino-Japanese football policies. It not only presents the differences between the two countries’ football policies but also reveals the underlying reasons from the perspectives of policy instrument theory and governance logic. However, there are certain limitations to analyzing Sino-Japanese football policies using the PMC index model. Binary scoring assumes all secondary variables are of equal importance, which may not reflect real-world policy priorities. Future research should explore weighted PMC models based on expert surveys or empirical outcomes (e.g., World Cup qualification). The main conclusions are as follows.

1. Through text analysis of 31 Sino-Japanese football policies, the study finds that the high-frequency words in China’s 18 policies are dominated by “campus, construction, management, reform,” which reflects China’s emphasis on football popularization and adopts a government-led, top-down governance logic. The high-frequency words in Japan’s 13 policies are dominated by “JFA, players, coaches, leagues, World Cup,” which reflects Japan’s emphasis on the competitive level of football and adopts an association-led, market-driven, competition-centered development logic. In the analysis of policy instrument mix, 6 football policies respectively from China and Japan were selected for analysis. China relies heavily on capacity-building instruments (50%) and authoritative instruments (25%), indicating a bias toward “strong top-level design, weak implementation.” Japan has a higher proportion of capacity-building instruments (73%) and fewer authoritative instruments (14%), reflecting a “strong capacity-building, weak administrative constraint” approach.
2. While the 12 Sino-Japanese football policies are both highly scientific and rational, there remains room for improvement. The average PMC index of all these policies is 7.59. China’s average PMC index (8.50) is higher than Japan’s (6.74). This difference is not accidental but a direct reflection of the two countries’ sports governance systems, power structures and development philosophies, rather than a simple difference in policy content. China’s football development is government-led, while Japan’s is association-led. The different sports governance models determine the orientation, instrument mix and implementation paths of the two countries’ football policies.
3. CP3 has a PMC index of 9.15, the highest among the six Chinese policies, featuring systematic and comprehensive content. JP_2_ has a PMC index of 7.77, the highest among the six Japanese policies, emphasizing close links and systematic synergy among the state, society, families and individuals. CP_1_ has a PMC index of 7.89, the lowest among the Chinese policies, with deficiencies in unclear responsible entities, vague reward and punishment measures, and unfocused competition goals. JP_6_ has a PMC index of 5.47, the lowest among the Japanese policies, with shortcomings in primary variables such as X_8_ (Policy Clarity) and X_2_ (Policy Timeframe).

Based on the above conclusions, the following policy recommendations are proposed.

1. Optimize the combination of policy instruments to enhance overall policy effectiveness.

Japan’s football development is led by the Football Association. Its policies are practical and flexible but lack sufficient administrative binding force in resource coordination and cross-sectoral promotion, leading to slow implementation. China’s policies are mainly issued by government departments, with high proportions of capacity-building instruments (50%) and authoritative instruments (25%). While China’s strengths lie in strong administrative coordination and resource allocation capabilities, it tends to adopt a one-size-fits-all approach. With the adjustment of the Chinese professional football league and the establishment of the Chinese Professional Football League, China’s football reform has entered a critical period of system restructuring. It is necessary to optimize policy instrument combinations, reduce administrative directives, increase the use of incentive instruments (10%) and system-reforming instruments (4%), and fully leverage the comprehensive effectiveness of policies.

2. Remedy existing policy shortcomings to enrich the content of policy instruments.

The PMC indices of these policies are positively correlated with the tertiary variable assignments of evaluation indicators. Strategic thinking should be adopted to focus on the overall situation, expand the coverage of policy content, and thereby enhance policy scientific validity and rationality. Among the 12 Sino-Japanese football policies analyzed using the PMC index model, Japan’s football policies consistently take claiming the World Cup as their long-term development objective, while China’s football policies lack the long-term planning objective for participating in the World Cup. Table 9 shows China’s X4 (Policy Objectives) mean value is 0.63, with zero policies mentioning World Cup participation (X_4−4_ = 0). Therefore, China should add explicit World Cup qualification targets to its medium- and long-term plans.

3. Enhance the intensity of international policy transfer and dialectically weigh the merits and demerits of policies.

Japan has a high proportion of capacity‑building instruments (73%) with a mature implementation mechanism. To optimize Chinese football policies, China should base itself on national conditions, broadly draw on foreign experience, and promote the leapfrog development of football. By learning from Japan’s J. League system and its community‑oriented philosophy [34], China can optimize the league structure, expand participation and enhance regional integration. By adopting Japan’s coach training and assessment strategies, coaching quality can be improved. In youth development, China can follow Japan’s dual focus on mass participation and elite development, building a three‑tier youth training system integrating schools, clubs and local football associations.

## Supporting information

S1 File Supporting information. (RAR)

S2 File Raw images. (PDF)
