# Correction: The comparative analysis of Sino-Japanese football policies from the perspective of policy instruments

**DOI:** 10.1371/journal.pone.0362103

**Published:** 2026-11-03

**Authors:** Chuanmin Zhang, Zanying Hu, Huqiang Wei, Mei Peng, Fei Gao

Figs 1–6 were uploaded incorrectly. Please see the correct Figs 1 – 6 here.

In the Overall evaluation of 12 Sino-Japanese football policies subsection of the Quantitative analysis of 12 Sino-Japanese football policies based on the PMC index model, there is an error in the sixth sentence of the first paragraph. The correct sentence is: Here in Fig 6, there is an exception in the mean values for the primary variables X4 (Policy Objectives), where China’s mean value was slightly lower than Japan’s.

In the Evaluation of 12 Sino-Japanese representative football policies subsection of the Quantitative analysis of 12 Sino-Japanese football policies based on the PMC index model, there is an error in the first sentence of the first paragraph. The correct sentence is: By comparing the content of Table 7, Table 9 and Figs 2–5 this study conducts a comparative analysis of the 12 representative football policies in China and Japan.

**Fig 1:**
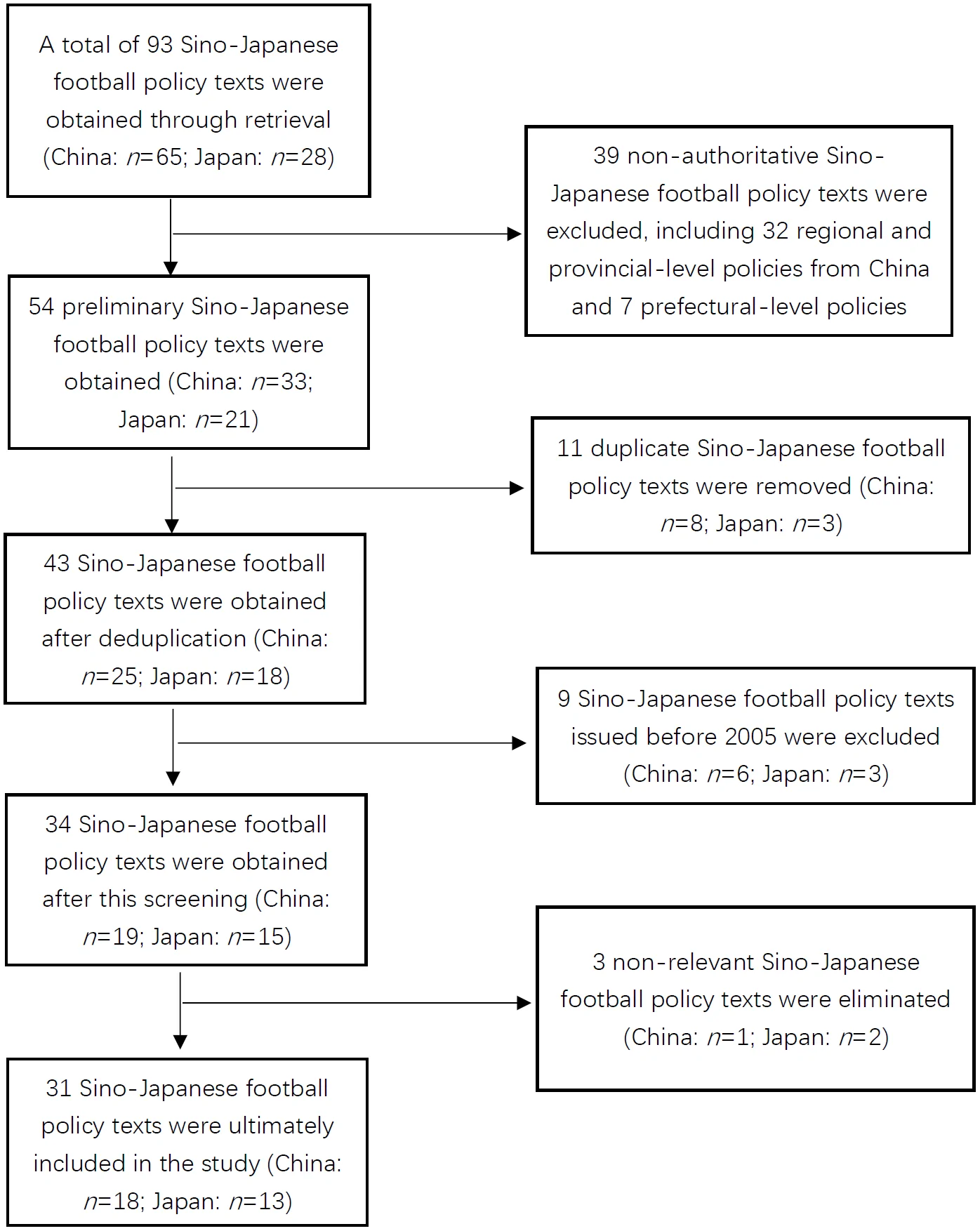
Sino-Japanese football policy screening flowchart.

**Fig 2:**
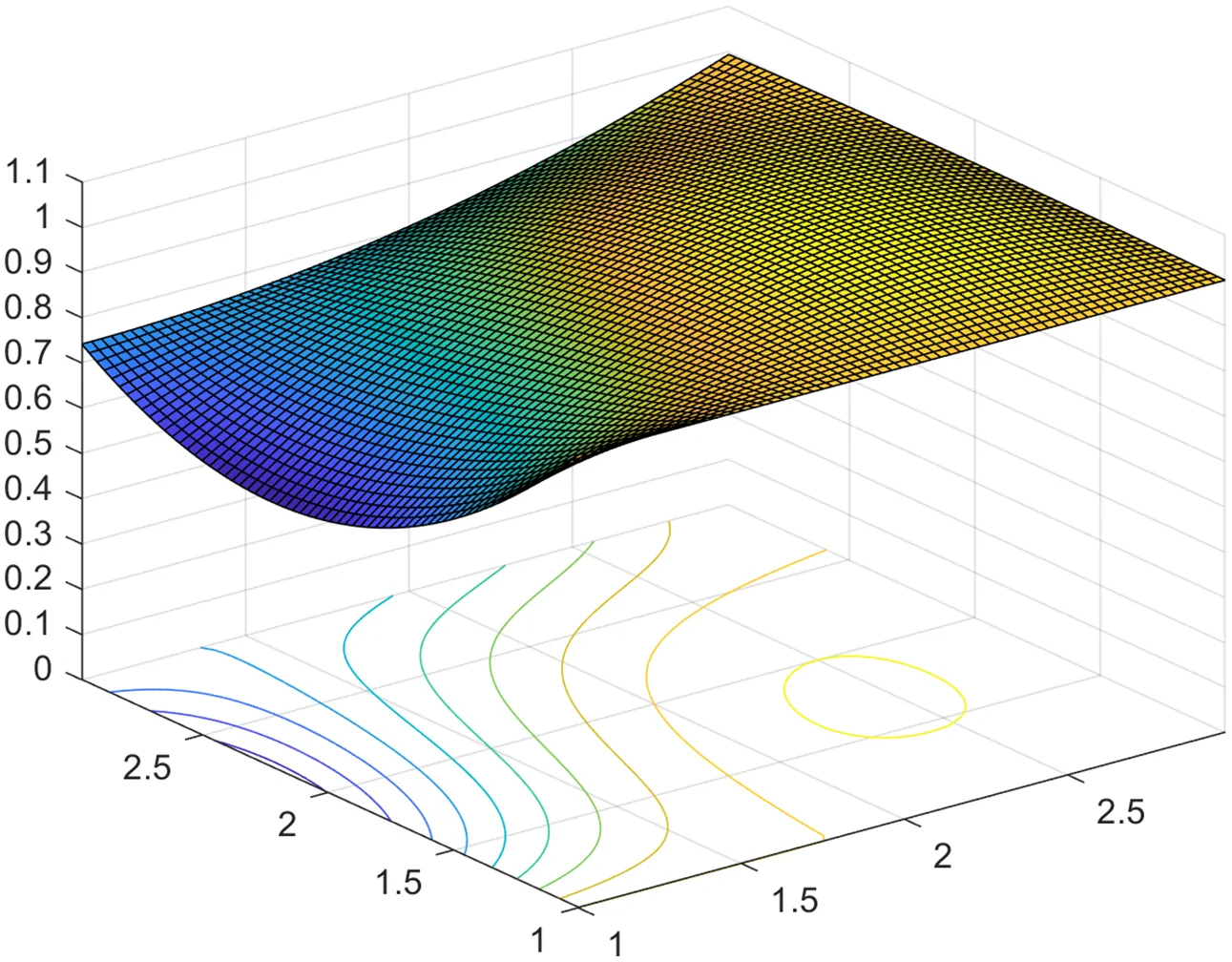
CP_3_ surface plot.

**Fig 3:**
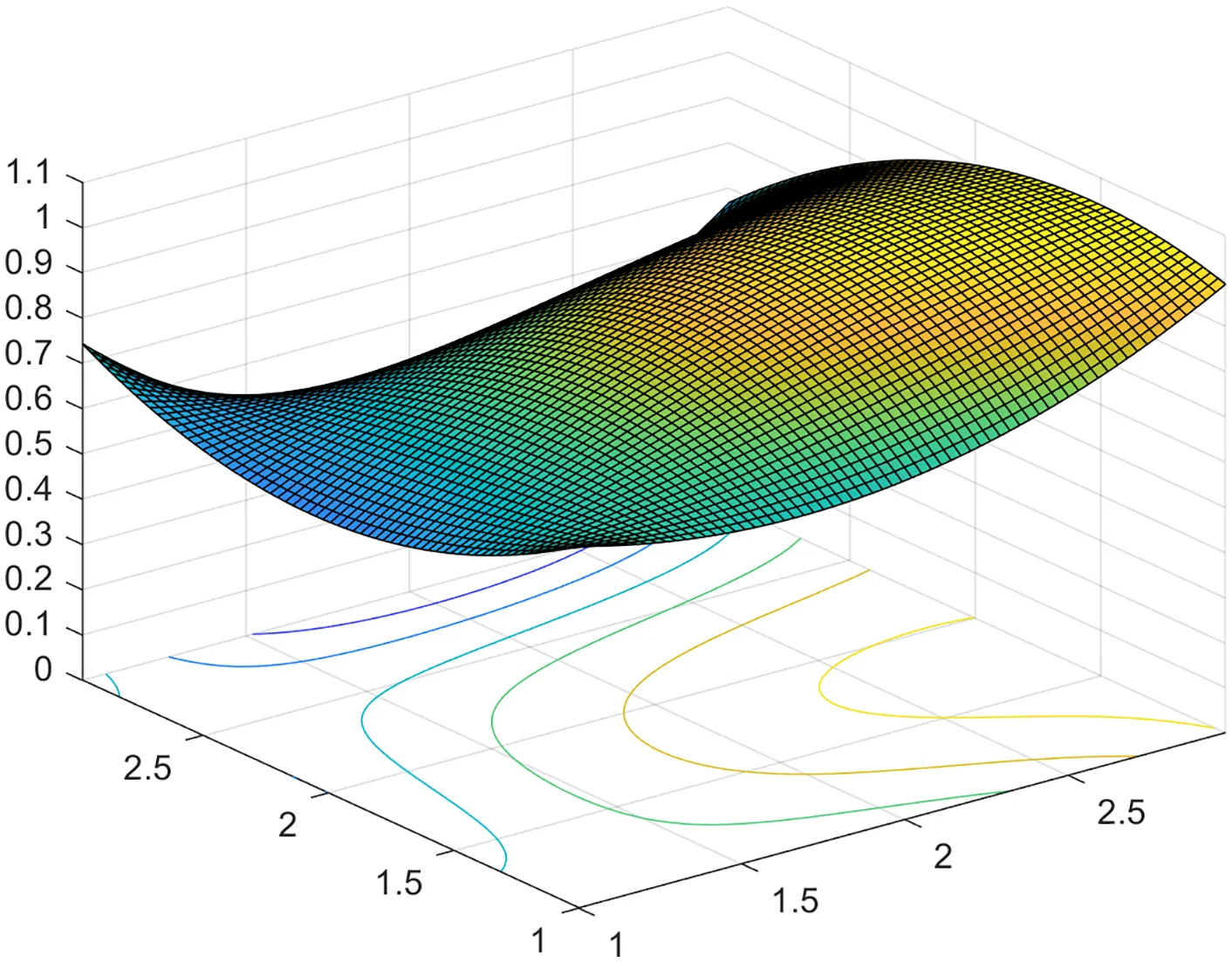
CP_1_ surface plot.

**Fig 4:**
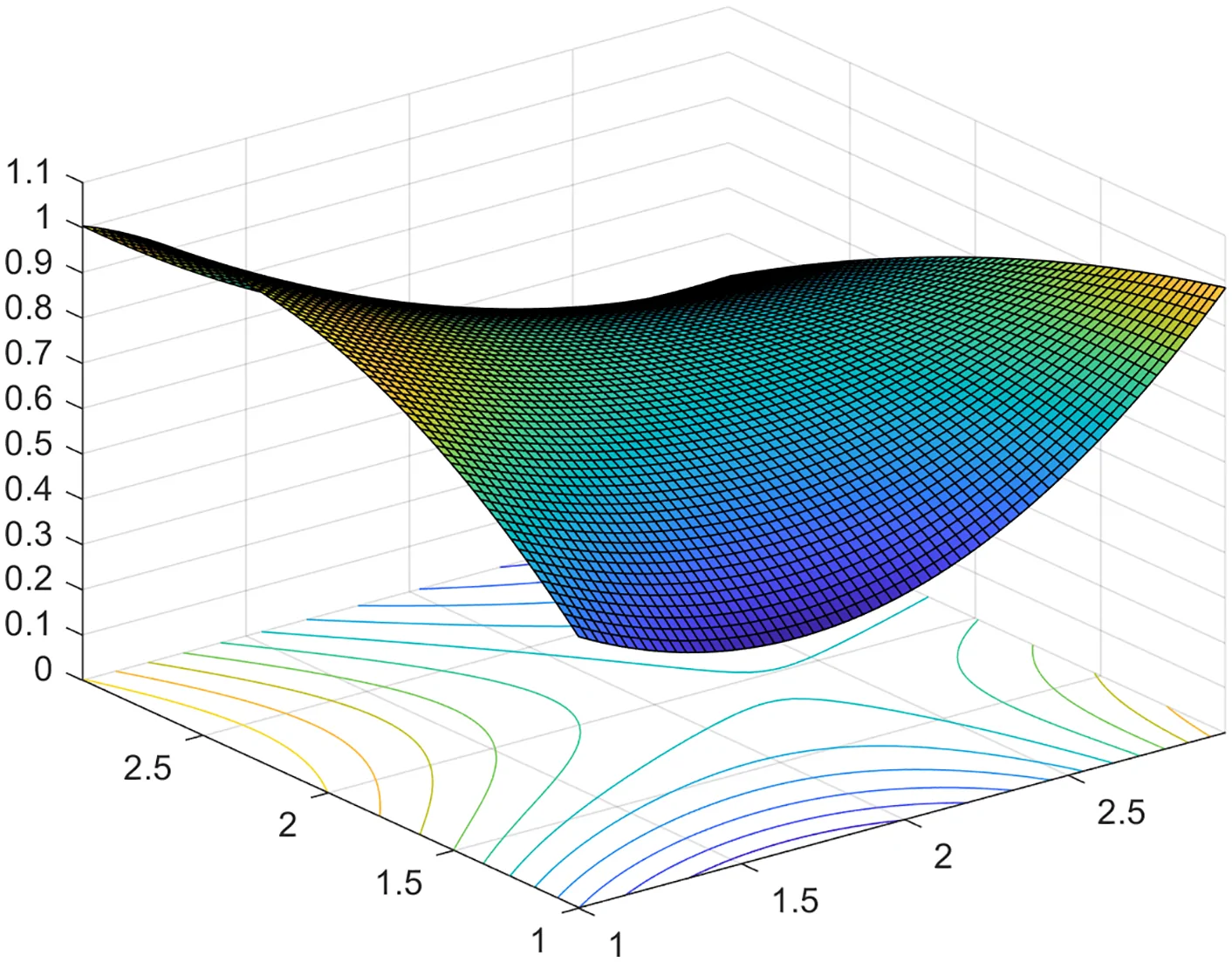
JP_2_ surface plot.

**Fig 5:**
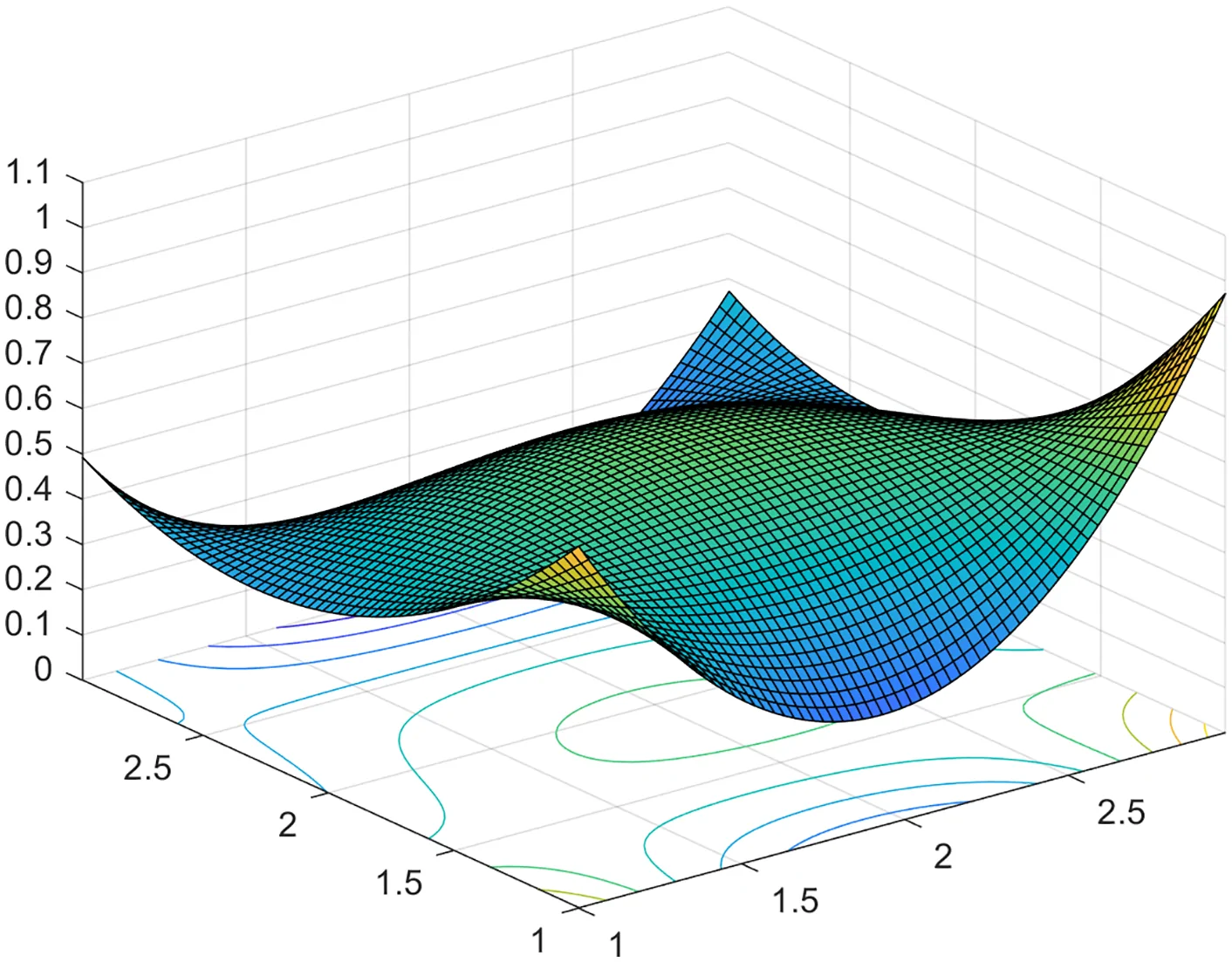
JP_6_ surface plot.

**Fig 6:**
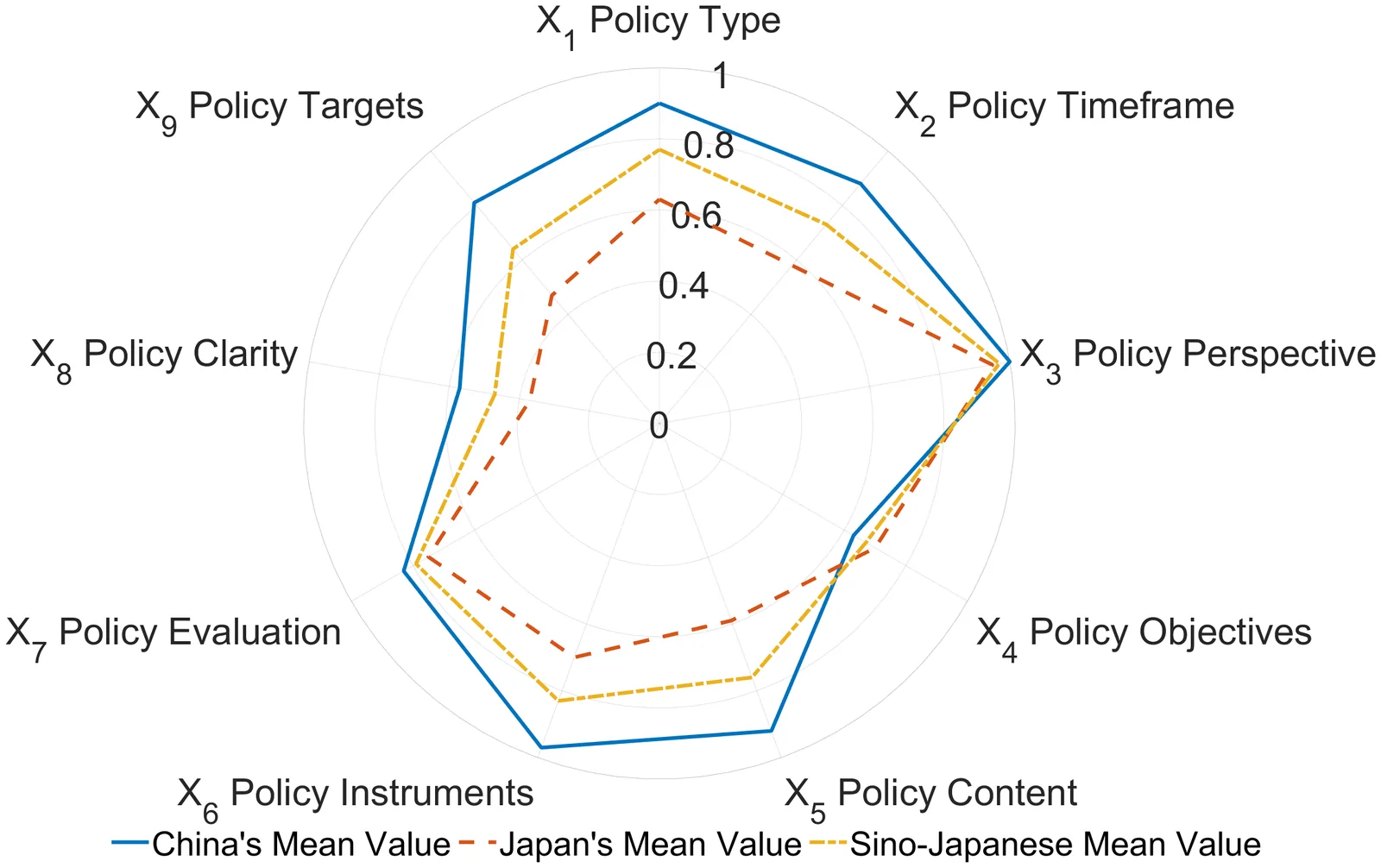
PMC Index mean value radar chart Sino-Japanese.
