# Supplementary material for: The comparative analysis of Sino-Japanese football policies from the perspective of policy instruments
Source: PLoS One. 2026 Jul 29;21(7):e0354667. doi: 10.1371/journal.pone.0354667 (PMC13421760; doi:10.1371/journal.pone.0354667)
Supplement: S2 File — (PDF) [file pone.0354667.s002.pdf]

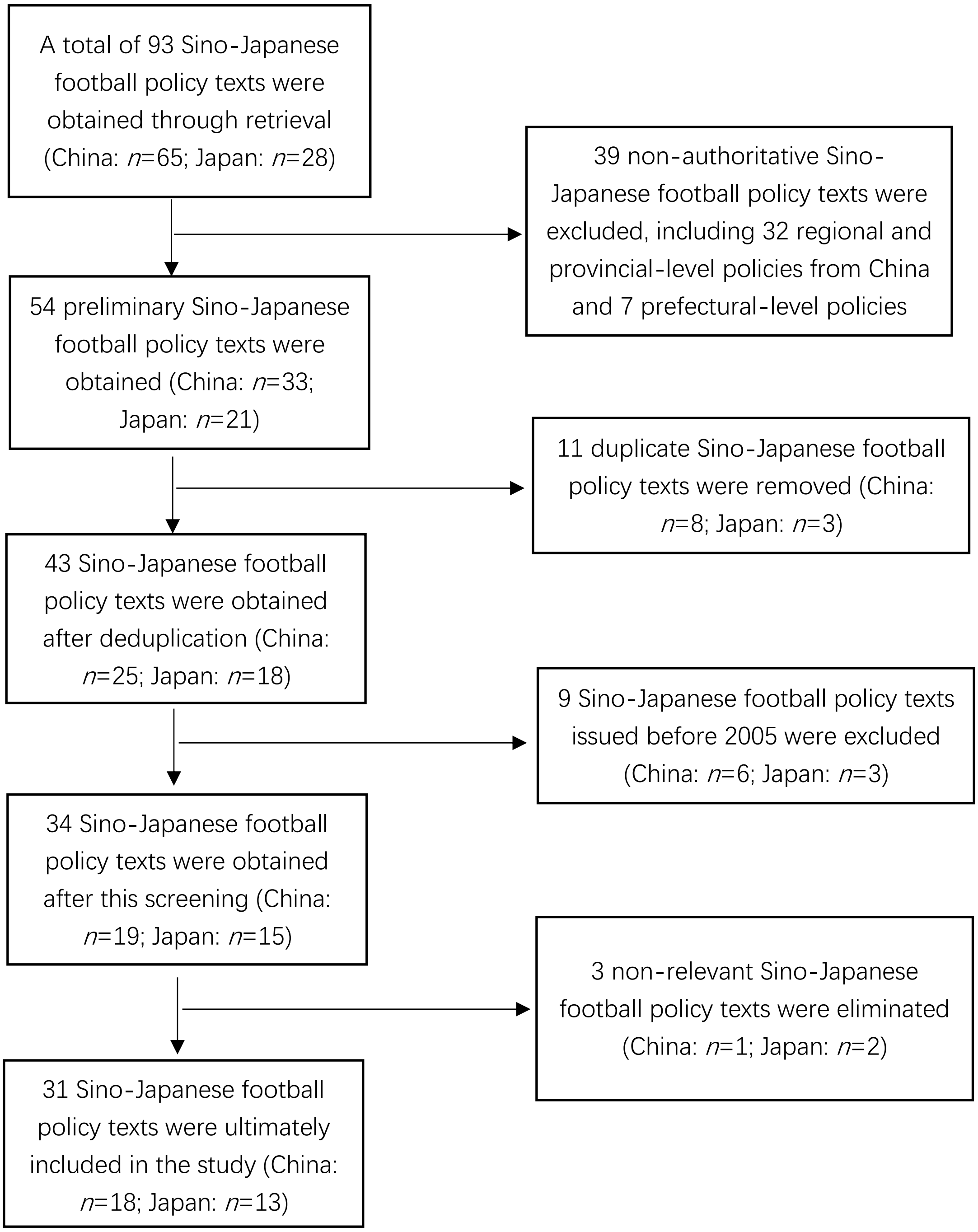

Figure 1 Sino-Japanese Football Policy Screening Flowchart

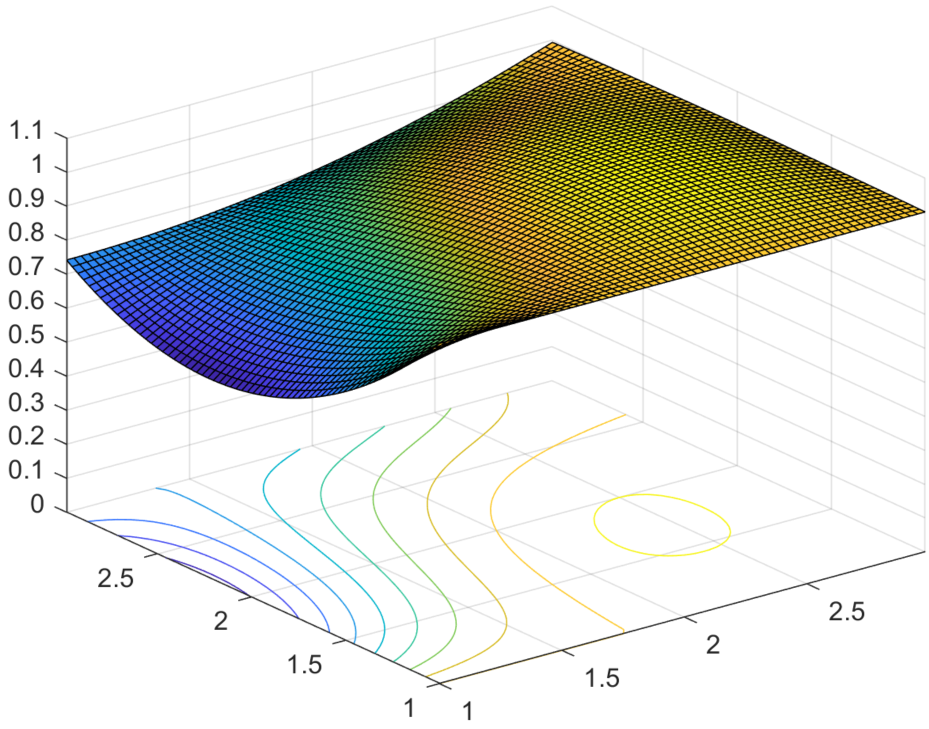

Figure 2. CP3 Surface Plot.

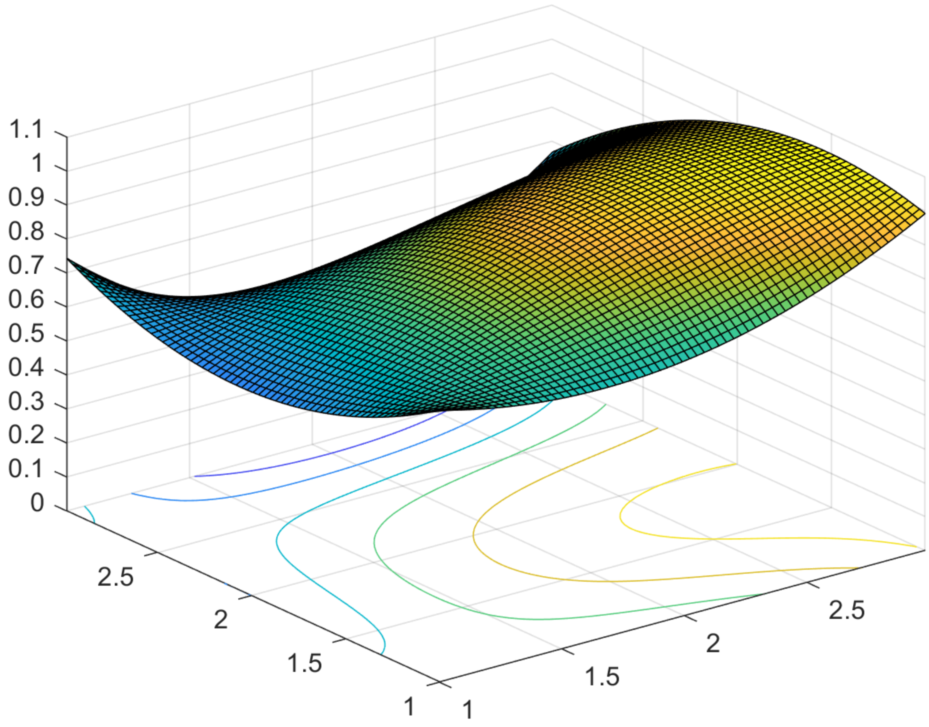

Figure 3. CP1 Surface Plot.

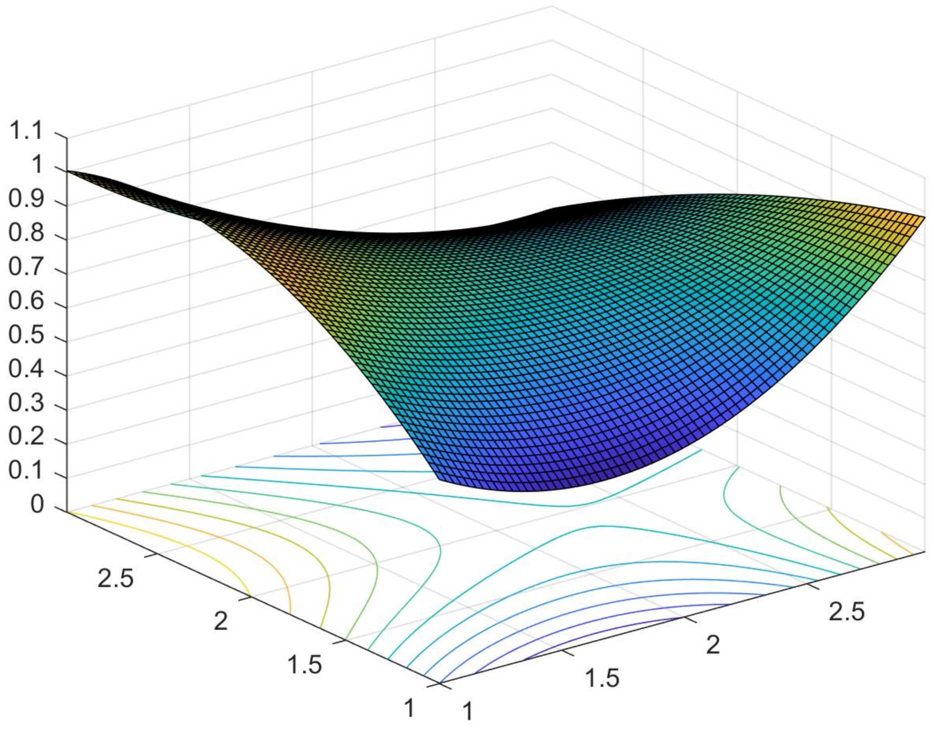

Figure 4. JP2 Surface Plot.

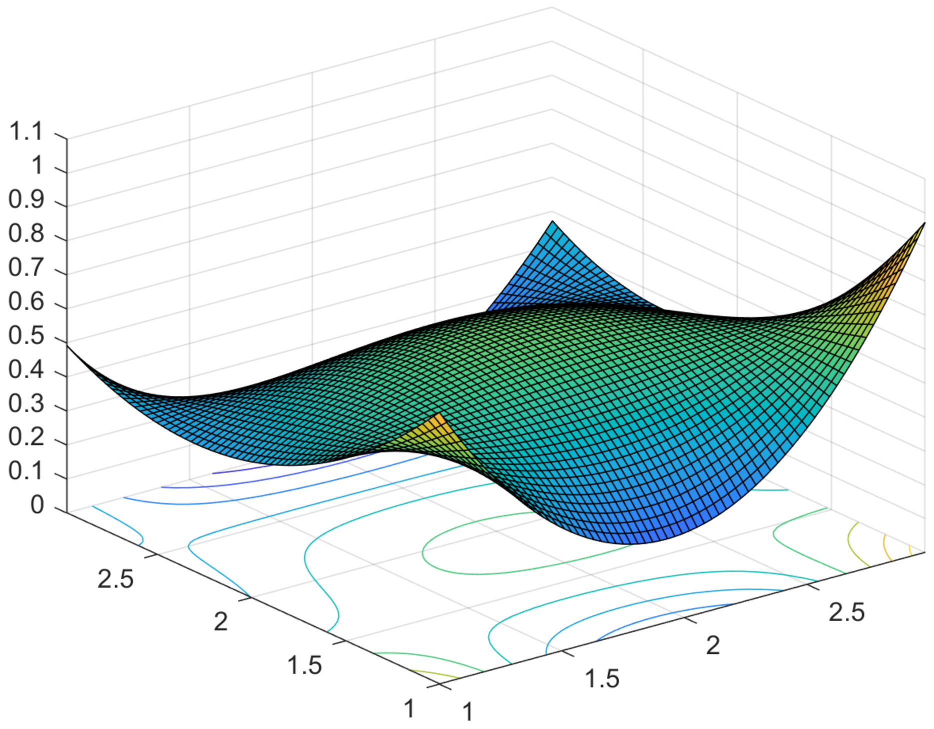

Figure 5. JP6 Surface Plot.

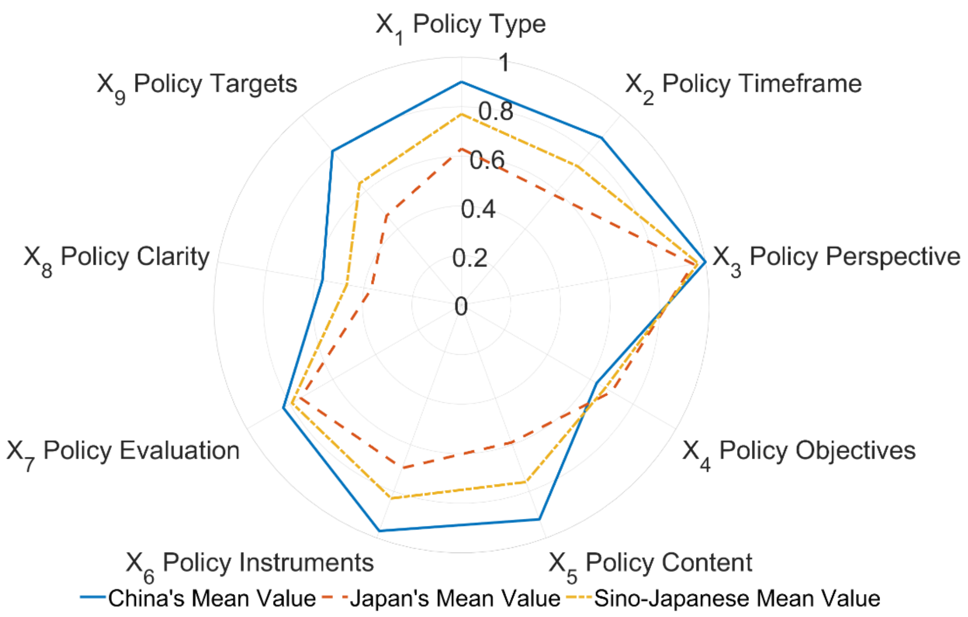

Figure 6. PMC Index Mean Value Radar Chart Sino-Japanese
