# Supplementary material for: The comparative analysis of Sino-Japanese football policies from the perspective of policy instruments
Source: PLoS One. 2026 Jul 29;21(7):e0354667. doi: 10.1371/journal.pone.0354667 (PMC13421760; doi:10.1371/journal.pone.0354667)
Supplement: Attachment — Submitted filename: Response to Reviewers.docx [file pone.0354667.s003.docx]

**Response to Reviewers**

Reviewer #1:

1. The English in the article needs improvement;

Response: Thank you for your reminder, Reviewer. We have carefully optimized and improved the English expression throughout the manuscript, corrected the non-standard and incoherent expressions, and strived to make the English expression accurate, rigorous and fluent to better present the research content.

2. The summary is confusing;

Response: We fully agree with your judgment. We have completely rewritten, revised and improved the abstract section, sorted out the logical context, optimized the language expression, to ensure that the core viewpoints of the abstract are clear, logically coherent and hierarchical, and accurately summarize the research focus and core conclusions of the entire manuscript.

3. Not only football policies can be a factor in change and sports development, but sports policies in general. If only football needs to be further exemplified;

Response: Thank you for your professional advice. We have deleted the statement "football policies play an important role in football reform and development" in the abstract to effectively avoid ambiguity, ensure more rigorous expression, and conform to your guiding opinions.

4. The methodological proposal seems innovative. On the other hand, there is a lot of information and the reader gets lost in the quantity and complexity of the data presented. We suggest that the authors develop a more intuitive data presentation proposal that actually compares the policies of different countries;

Response: We sincerely thank you for your suggestion. We have simplified the relevant descriptions in Section 3.2.4 of the manuscript. Regarding the data presentation form, presenting word frequency, multi-input-output scale and PMC index table in the form of tables is a conventional method in PMC policy analysis. For example, in the following papers: 1. Hong S, Wang T, Fu X, Li G. Research on quantitative evaluation of digital economy policy in China based on the PMC index model. Peng J, editor. PLOS ONE. 2024;19: e0298312. doi:10.1371/journal.pone.0298312; 2. Cui C, Wang K. Quantitatively analyzing the college student employment policy in China based on PMC-index model. Cobo-Martín MJ, editor. PLOS ONE. 2024;19: e0310479. doi:10.1371/journal.pone.0310479, such table forms are used. Therefore, this form is still retained in this revision, and we sincerely request your understanding.

5. The conclusion in bullet points provides what one would expect from a discussion of the results, leaving out an actual conclusion of the text.

Response: We deeply recognize the importance of this issue. We have completely rewritten and revised the conclusion and suggestion sections, making the conclusion section more concise and concise, highlighting the key points, and fully reflecting the summarization and generality of the entire research; at the same time, we have optimized the suggestion section, closely combining the research results and core findings of the manuscript to enhance the pertinence and relevance of the suggestions, ensuring that they meet the normative requirements of conclusions and suggestions in academic papers.

We would like to express our sincere gratitude to you again for your careful guidance and professional advice. We have made corresponding revisions in accordance with all the suggestions you put forward. Although we have strived for perfection, there may still be omissions. We sincerely request you to give further corrections. We will attach great importance to your subsequent opinions, promptly and earnestly make improvements and revisions, and make every effort to improve the quality of the manuscript, living up to your guidance and expectations.

Reviewer #2:

1.The authors could strengthen the introduction by examining football policies more broadly, worldwide, including in Asia and neighboring countries, and then exploring the policies of China and Japan.

Response: Thank you for your valuable suggestion. We have added relevant literature in the introduction to broaden the research perspective, including: the impact of anti-hooligan policies on football match ticket sales, football club transfer policies, the impact of policies prohibiting the sale of alcoholic beverages in football stadiums on crime rates, the impact of economic control policies on the finances of professional football clubs, and the impact of football policies on football match safety.

2.The PMC results show that Policy Clarity is a consistent weakness for both nations. However, the paper fails to critically examine whether this lack of clarity is a strategic choice or a governance failure, particularly in the Chinese context, where macro-level direction is often prioritized over operational details.

Response: We deeply agree with your judgment. We have added relevant content in Section 4.5.1 of the manuscript: "As indicated in mean values of primary variables, both China and Japan achieved low values in policy clarity. In China’s case, inadequate policy clarity was mainly ascribed to the top-down design and hierarchical implementation of its sports policies, which tended to result in ambiguous definitions of specific details. For Japan, the absence of policy clarity in football policies was attributed to its market-led policy framework, so that, in formulating the policies, it cannot better balance the demands of multiple entities, including football associations, professional league bodies, clubs, and local governments, thereby resulting in ambiguous policy statements."

3.While the MATLAB-generated surface plots are visually impressive, they largely confirm what is already in the tables. The paper does not sufficiently explain how these visualizations provide a unique analytical advantage over raw PMC index scores.

Response: Thank you for your professional suggestion. We have added " The PMC surface plot can matrix the first-level variable values of each policy, and the advantages and disadvantages of the policy can be directly observed through the surface plot." in Section 4.4 of the manuscript, and added the following content in Section 4.5.2: " As illustrated by Table 8 and Figure 1, CP3 yields the highest PMC index among Sino-Japanese football policies."; " It can be seen from Figure 2 that the policy exhibits a relatively significant depression in X_8_ (Policy Clarity)."; " It can be seen from Figure 3 that JP2 shows certain depressions in X_2_ (Policy Timeframe) and X_9_ (Policy Targets), indicating that the policy not only lacks short-term and ultra-short-term plans but also has an unclear division of responsibilities between government departments and clubs."; " It can be seen from Figure 4 that the policy has obvious deficiencies in X_2_ (Policy Timeframe), X_4_ (Policy Objectives), X_6_ (Policy Instruments) and X8 (Policy Clarity), because the policy ignores specific implementation plans and details."

4.The Conclusion section is too long and does not constitute a genuine scholastic paper conclusion. The conclusion section should succinctly summarize the key findings, highlight the main takeaways, and provide actionable recommendations for policy improvement. This factual separation from the discussion would make the paper more organized and ensure that the conclusions are presented in a focused and impactful manner, in a short, coherent, and meaningful paragraph.

Response: Thank you for your careful guidance. We have improved the relevant content in strict accordance with your requirements. On the one hand, we have targeted deleted and refined the conclusion section, abandoned redundant expressions, briefly and concisely summarized the main research findings of the entire manuscript, highlighted the core points, and strived to present the conclusion in a short, coherent and powerful paragraph; on the other hand, we have revised and improved the suggestion section, put forward executable policy improvement suggestions focusing on the core research findings, and clearly distinguished the content boundary between the conclusion and the discussion section, further sorted out the manuscript structure, to ensure that the logic of the manuscript is clearer, the expression is more rigorous, and it conforms to the normative requirements of the conclusion of an academic paper.

5.The authors should discuss the structural differences between China’s government model and Japan's model. Explaining how these different issuing authorities can affect policy implementation would add significant depth.

Response: Thank you for your careful review and professional advice. We have added relevant content in Part 1 of the suggestions: Football policies promulgated by JFA are characterized by their practice orientation and flexible adaptation, but are lacking in adequate administrative binding force in resource coordination and cross-domain advancement, resulting in sluggish policy implementation. China’s football policies are issued by the government departments. Their advantages are embodied in their strong administrative coordination and resource allocation capabilities, while their disadvantage is the great tendency to lead to a one-size-fits-all situation.

6.The recommendation to promote stakeholder diversification is vague. The paper should provide concrete examples of how China could adopt Japan’s three-pronged approach (Coach, youth, and National team cohesiveness) within a specific regulatory framework.

Response: Thank you for your professional suggestion. We have added relevant content in Part 3 of the suggestions: Through incorporating Japanese strategies of cultivating and assessing coaches, we can progressively nurture female coaches from sports colleges and grassroots sectors, constantly increase the number of registered coaches, and enhance their teaching capacity and competence through continuing education and professional training. Notwithstanding China’s introduction of many robust supportive policies from the state level, football is not the most widespread and the most preferred sport among children and youth. Accordingly, in terms of youth’s training, we should draw on Japan’s football model of equal emphasis on popularization and elite to advance the youth training system that integrates campuses, clubs and local football associations. In terms of the cohesiveness of national teams, we should learn from Japanese culture-led strategies to positively publicize national teams’ competitive spirit, reinforce their ideological and cultural construction, and foster their spirit of solidarity, striving and unyieldingness, thus collectively promoting the sound, scientific and sustainable development of Chinese football cause.

7.While the paper identifies that China lacks focus on World Cup participation targets in its policies compared to Japan (the author may put the results of the Japan team in the previous World Cups), it should explore why (e.g., differing philosophies of long-term vs short-term success) to provide more actionable ‘international benchmarking.

Response: Thank you for your suggestion. We have added the following content in Section 3.5.1: Japan Men's National Football Team has qualified for seven consecutive World Cups since their debut in the 1998 tournament in France, and progressed beyond the group stage on four occasions—in 2002, 2010, 2018, and 2022. Thereby, five of Japan’s six football policies explicitly mentioned World Cup participation objectives.

We have added the following content in Part 2 of the suggestions: Japan’s football policies consistently take claiming the World Cup as their long-term development objective, while China’s football policies lack the long-term planning objective for participating in the World Cup.

8.The authors could add to the Discussion that football is neither the most common nor the preferred sport or physical activity among children and students, despite strong government policies.

Response: Thank you again for your careful guidance. We have added the following content in Part 3 of the suggestions: Although strong support policies have been introduced at the national level, football is not the most popular or favored sport among children and adolescents.

We have revised the relevant suggestions you put forward. If there are any inappropriate parts, we sincerely request further corrections. We will make timely improvements to ensure the research quality.
