# Supplementary material for: The comparative analysis of Sino-Japanese football policies from the perspective of policy instruments
Source: PLoS One. 2026 Jul 29;21(7):e0354667. doi: 10.1371/journal.pone.0354667 (PMC13421760; doi:10.1371/journal.pone.0354667)
Supplement: Attachment — Submitted filename: Response_to_Reviewers_auresp_2.docx [file pone.0354667.s004.docx]

PONE-D-25-49612R1

Response to Reviewers

Dear Academic Editor,

Thank you for the constructive and detailed comments on our manuscript. We have carefully revised the paper according to each point raised. Below is a point-by-point response, followed by a summary of major revisions made in the manuscript.

**1. Reviewer’s comment: The manuscript needs clearer concepts, stronger methods, and deeper analysis. The theoretical contribution is not clear enough, as the study is mostly descriptive and does not show how it fits into the wider international sport policy literature.**

**Response:** We fully agree and have made substantial revisions.

1.1 Added an international literature review in the Introduction (Pages 2). We cited international studies on policy instrument theory and PMC model applications in sports policy evaluation, clearly positioning our work in the international sport policy literature.

1.2 Added a “Theoretical Framework” section (Page 3, 6). We explicitly state that this study is based on policy instrument theory (McDonnell, 1987) and PMC index model (Ruiz Estrada, 2011), and clarify its contribution: the first systematic PMC-based comparison of Sino–Japanese football policies, filling a gap in Asian sport policy research.

**2.Reviewer’s comment: The way policies were chosen is not transparent and could be biased, since terms like “authority” and “relevance” are not clearly defined, and the difference in the number of Chinese and Japanese policies is not explained.**

**Response:**

2.1 We have clarified all selection criteria and explained the numerical difference.

Defined policy selection criteria (Page 3):

Authority: Official documents issued by national government agencies or national football associations.

Relevance: Core content on football development/reform/planning.

Timeliness: Policies issued between 2005–2025.

Explained the number difference (Page 3):

2.2 China: 18 policies (multi-departmental, multi-level government-led system).

Japan: 13 policies (JFA-dominated, fewer government documents).

**3. Reviewer’s comment: The coding process, even though it reports high reliability (p. 16), does not give enough detail about how coding was done or how disagreements were handled.**

**Response:** We added detailed coding procedures.

4.1Added coding workflow and reliability check (Page 7-8):

Two coders independently coded all texts.

Disagreements were resolved through discussion; unresolved items were arbitrated by a third expert.

Kappa values >0.87 confirmed high reliability.

**4. Reviewer’s comment: Another concern is the use of the PMC index model (pp. 20–22). Using binary scoring and equal weighting makes complex policy content too simple and is not supported by theory, which affects how valid the findings are.**

**Response:** We acknowledge the limitation and provided theoretical justification.

（pages 3, 12）, we point out that the PMC model is widely adopted internationally, and that binary assignment weights can ensure objectivity and comparability. Relevant references have also been added as evidence.

**5.Reviewer’s comment: The results are mostly described without much critical analysis or theoretical insight, and some conclusions, especially policy recommendations, seem more like opinions than findings based on the data.**

**Response:** We strengthened data-based analysis and evidence-based recommendations.

5.1 All conclusions are now supported by PMC scores, coding frequencies, and indicator values (Pages 21-22).

5.2 Every recommendation is linked to specific data findings (Page 22-23).

**6. Reviewer’s comment: The comparison between China and Japan is also not deep enough, as it does not look at important background factors like governance or political systems that could explain the differences.**

**Response:** In the first paragraph of the conclusion（page 22）, we have added an analysis of key contextual factors such as the governance models and political systems of China and Japan.

China: Government-led, multi-level administrative governance.

Japan: JFA-led, market-oriented, community-based governance.

**7. Reviewer’s comment: Finally, while the manuscript says all data are available, it does not include the raw coding outputs or NVivo data, which makes it hard to reproduce the study.**

**Response:** We have provided full supplementary materials.

8. Reviewer’s comment: Besides these main issues, there are also some smaller problems with clarity and presentation…

Response: We have revised thoroughly:

We have invited native English-speaking scholars to revise unclear expressions and grammatical issues, correcting grammatical errors, standardizing terminology, and improving readability. Regarding the table issue, we need to state that existing articles on PMC policy analysis all adopt this table format for statistical presentation. This tabular format is a conventional method in PMC policy analysis and has been widely accepted and adopted in the field (Hong S, 2024). Therefore, in this study, we have continued to use this standardized presentation to ensure consistency of methods and comparability of results. We kindly ask the editor for understanding and approval.

Thank you again for your valuable comments. The revised manuscript has been significantly improved in theoretical depth, methodological rigor, data transparency, and readability. We hope the revised version meets the journal’s standards.

Sincerely,

Corresponding Author

Fei Gao
