# Supplementary material for: The comparative analysis of Sino-Japanese football policies from the perspective of policy instruments
Source: PLoS One. 2026 Jul 29;21(7):e0354667. doi: 10.1371/journal.pone.0354667 (PMC13421760; doi:10.1371/journal.pone.0354667)
Supplement: Attachment — Submitted filename: Response_to_Reviewers_auresp_3.docx [file pone.0354667.s005.docx]

**Dear Editor,**

Thank you very much for your valuable comments and suggestions on our manuscript. We sincerely appreciate the time and effort you have dedicated to reviewing our research. Your insightful feedback has significantly improved the quality and clarity of the manuscript.

**Comment** 1: Page 2, line 31 and Page 3, line 10.

The authors mention adding a "Theoretical Framework" section in their response to reviewers, but in the manuscript file, this section is not clearly labeled or separated from the introduction. The theoretical basis (McDonnell & Elmore, 1987; Ruiz Estrada, 2011) is mentioned only in passing within the introduction and methods. Create a clear, labeled Section 2.0 "Theoretical Framework" before Section 2.1. Explicitly state: How policy instrument theory (McDonnell) structures your coding scheme (Table 3). Why the PMC index model (Ruiz Estrada) is appropriate for comparing sports policies across different governance systems (China's state-led vs. Japan's association-led). Your theoretical contribution: e.g., "This study integrates policy instrument theory with quantitative PMC modeling to explain how governance systems shape policy instrument mixes in non-Western sport contexts."

**Response 1:** We have added the relevant content on page 3, lines 76-97 (Section 2.1).

This study integrates policy instrument theory and the PMC index model to construct a two-dimensional analytical framework for policy instrument typology and quantitative evaluation of policy quality. Policy instrument theory categorizes policy implementation instruments into five types [18]: authoritative, incentive, capacity-building, systemic reform, and symbolic and hortatory, providing operational coding dimensions for this study. Based on this theory, Table 3 further refines 18 third-level nodes, transforming the content analysis of policy texts from subjective description to structured classification and effectively revealing the preferences and structural imbalances in instrument selection between China and Japan. Meanwhile, the PMC index model, as a quantitative policy evaluation tool, adopts a binary assignment method without preset variable weights, enabling objective comparison of policy quality under different governance systems. This model is particularly suitable for analyzing the two significantly different sports governance models: the "state-led" model in China and the "association-led" model in Japan, where the former emphasizes administrative coordination and institutional design, while the latter relies on market-driven mechanisms and industry self-regulation. The comparison between the two cannot be limited to content differences but requires quantifiable comparison under the same evaluation framework. Based on the above theoretical integration, the core contribution of this study is: This study integrates policy instrument theory with quantitative PMC modeling to explain how governance systems shape policy instrument mixes in non-Western sport contexts.

**2. Policy selection criteria remain ambiguous–especially "authority" and "relevance."**

**Comment 2: Page 3, lines 20-25.**

The authors define "Authority" as "official documents from national governments or football associations" and "Relevance" as "core content on football development/reform/planning." However, it is unclear what constitutes "core content" (e.g., minimum percentage of text? specific keywords?). Provide an operational definition for each:

- Authority: List the exact issuing bodies accepted (e.g., State Council, General Administration of Sport of China, JFA, MEXT). Exclude regional or city-level policies.

- Relevance: A policy is "relevant" if the word "football" appears in the title or in at least 20% of the main text. State this explicitly.

Add a flow diagram in the supplementary materials showing the inclusion/exclusion process with numbers.

**Response 2:** We have added the relevant content on pages 3 and 4, lines 107-116 (Section 2.2). Policy authority and standardization refer to official documents issued by the General Office of the State Council, the General Administration of Sport of China, and the JFA. All regional and provincial policies in China, as well as all prefectural policies in Japan, are excluded from the sample. Relevance means that the policy mainly involves football development, reform, planning and other related content, and the word "football" appears at least 10 times in the policy text.

In addition, we have added the Sino-Japanese football policy screening flowchart and uploaded it as an attachment.

**Comment 3: Page 3, lines 22-25.**

The explanation ("China is multi-departmental, Japan is JFA-dominated") is insufficient. This difference could bias the PMC comparison because more policies allow China to potentially score higher on aggregated metrics. Explicitly state that the comparative PMC analysis is only performed on the 12 matched policies (6 from each country) – not all 31. Explain how those 6 were selected from each national set (e.g., by time period, by policy type, by purposive sampling for comparability). Justify why the other 22 policies were excluded from the quantitative model.

**Response 3:** We have added explanations of the relevant content on page 4, lines 134-137 (Section 2.4).

To ensure the comparability of Sino-Japanese football policies, the purposive sampling method was adopted to select 6 policies each from China and Japan related to overall football development planning, children and youth football development, and women's football development for PMC index analysis.

Comment 4: Page 7-8, lines 173-183 (section 3.2.2).

The authors report Kappa > 0.87 but do not describe how many disagreements occurred, what specific nodes caused disagreement, the qualifications of the "third expert" who arbitrated. Add a short paragraph or supplementary table:

"A total of 47 coding disagreements occurred across 592 reference points (7.9%). Disagreements were most frequent in the tertiary nodes 'Platform Construction' (n=12) and 'Functional Delineation' (n=9). The third expert was a professor of sport policy with 10 years of experience. All unresolved items were discussed in two consensus meetings."

**Response 4:** We have added the relevant content on pages 9 and 10, lines 254-259 (Section 3.2.2).

A total of 47 coding disagreements occurred across 592 reference points (7.9%). Disagreements were most frequent in the tertiary nodes 'Platform Construction' (n=12) and 'Functional Delineation' (n=9). The third expert was a professor of sport policies with 10 years of experience. All unresolved items were discussed in two consensus meetings.

**Comment 5: Page 12, lines 313-315; also page 3, lines 12-14 (response to reviewers).**

The authors defend binary scoring as "widely adopted" but do not address whether equal weighting is valid for football policies where some variables (e.g., funding) may be more important than others (e.g., ultra-short-term planning). Add a Limitation sub-section in the Discussion (around page 28-29) stating: "Binary scoring assumes all secondary variables are of equal importance, which may not reflect real-world policy priorities. Future research should explore weighted PMC models based on expert surveys or empirical outcomes (e.g., World Cup qualification)."

Also run a sensitivity analysis (as a supplementary file) where one or two key variables (e.g., X8 Policy Clarity, X4 World Cup objective) are given double weight, and report whether the ranking changes.

**Response 5:** We have added the statement of limitations on page 24, lines 631-635 (Section 5). We have also conducted the sensitivity analysis, which is presented as Supplementary File S2.

However, there are certain limitations to analyzing Sino-Japanese football policies using the PMC index model. Binary scoring assumes all secondary variables are of equal importance, which may not reflect real-world policy priorities. Future research should explore weighted PMC models based on expert surveys or empirical outcomes (e.g., World Cup qualification).

**Comment 6: Page 22-23, lines 528-557 (recommendations).**

The first recommendation ("Optimize policy instrument combinations") refers to China's 50% capacity-building and 25% authoritative instruments–good. But the second recommendation ("Remedy existing policy shortcomings") mentions "World Cup participation objectives" – however, Table 8 shows none of China's policies included World Cup goals (X4-4 = 0 for all CPs). The recommendation is correct, but the link to the data is not explicit. Restructure each recommendation as:

- Finding (data): "Table 8 shows China's X4 (Policy Objectives) mean value is 0.63, with zero policies mentioning World Cup participation (X4-4 = 0)."

"Therefore, China should add explicit World Cup qualification targets to its medium- and long-term plans."

**Response 6:** We have added the following content in the recommendations section on page 25, lines 699-702.

Table 8 shows China’s X4 (Policy Objectives) mean value is 0.63, with zero policies mentioning World Cup participation (X4-4 = 0). Therefore, China should add explicit World Cup qualification targets to its medium- and long-term plans.

**Comment 7: Page 22, lines 517-519.**

The authors contrast China's "government-led, multi-level administrative governance" with Japan's "JFA-led, market-oriented governance." This is critical context, but it appears only at the end. Move this comparison to a new sub-section 2.4 "Governance Context of Sino-Japanese Football" before presenting results. Then refer back to it when interpreting differences in policy instruments (e.g., why Japan uses fewer authoritative instruments).

**Response 7:** We have added Section 2.5 on pages 4 and 5, lines 138-170, to explain the governance background of Sino-Japanese football.

2.5 Governance Background of Sino-Japanese Football

Football governance in China is a national governmental act led by multi-departmental and multi-level governments. This is mainly because China's competitive sports adopt the nationwide system. As an important component of the sports cause, football has also followed this development path of government overall planning and administrative force promotion. The Plan, promulgated in 2015, was issued by the General Office of the State Council and jointly promoted by multiple ministries and commissions. In addition, football development involves multiple fields such as education and finance, and the government-led form can effectively integrate scattered public resources. This governance model is driven by administrative authority and achieves unified action nationwide through top-down policy deployment.

In contrast, football governance in Japan has formed an industry self-governance model led by the JFA and market-oriented. The JFA was founded in 1921 and possesses independent personnel, financial and policy-making powers. The Japanese government mainly plays a supporting role in football development and does not directly interfere in the internal affairs of the industry. This governance model is oriented by the endogenous needs of the industry and realizes development through market mechanisms and social self-governance.

The analysis of the governance background of Sino-Japanese football provides the institutional foundation for understanding the subsequent differences in the structure of football policy instruments and the divergence in quality characteristics between the two countries.

We have also mentioned the comparison of the governance background of Sino-Japanese football when explaining the differences in policy instruments in Section 3.2.4, on page 11, lines 302-304.

As stated earlier, it is precisely the different governance contexts of Sino-Japanese football that lead to the differences in the use of football policy instruments between the two countries.

**Table 1 comment**

Table 1 (keyword frequency) is difficult to interpret with no clear column alignment. The table splits "China" and "Japan" columns but the layout is confusing. Many rows are blank or misaligned. Reformat Table 1 as two separate tables (Table 1a for China, Table 1b for Japan) or use a landscape layout with clear vertical lines separating the two countries. Ensure all 40 ranks are visible.

**Response:** We have reformatted Table 1 into two separate tables (Table 1a for China, Table 1b for Japan)

**Table 8 comment**

A row labeled "Concavity Index" appears with numerical values (2.11, 1.37, etc.) but the concept is never defined in the text. Add a sentence in Section 4.3 or 4.4: "The Concavity Index measures the average deviation of the PMC surface from a flat plane; higher values indicate greater variability among primary variable scores." Alternatively, remove it if not interpreted.

**Response 8:** We have revised the relevant descriptions in Section 4.4, lines 442-445, to make the expressions clearer.

The deeper the concavity of the surface, the darker the cool tones of the surface and the base contour lines, which indicates a lower PMC index and a higher concavity index for the policy. Conversely, the stronger the warm tones of the surface and the base contour lines, the higher the PMC index and the lower the concavity index.

Typographical and grammatical errors comments

Page 1, line 34: "football's progress": should be "football's development" (repetitive).

We did not find the phrase "football's progress" on page 1, line 34, or anywhere else in the full text.

Page 12, line 44: "Overall Plan for Chinese Football Reform and Development [2] (hereinafter referred to as The Plan)": missing closing quotation mark or italics.

We have formatted all policy titles in italics.

Page 29, line 416: "five of Japan's six football policies": but Japan has 13 total and 6 in the PMC sample. Be specific: "five of the six Japanese policies in the PMC analysis."

We have revised the content on page 21, lines 536-537 as follows:

This is also why 5 out of the 6 football policies analyzed using the PMC index model mention World Cup participation goals.

Perform a full copy-edit focusing on consistent use of definite/indefinite articles, clear differentiation between "China's policies" (all 18) vs "the six selected Chinese policies.", standardized reference formatting (some URLs are broken, e.g., ref 2 on page 34).

We have revised the relevant content throughout the manuscript to ensure consistent use of definite and indefinite articles, and clarified the number of policies in the paper.

We have added the following content on page 24, lines 637-645:

Through text analysis of 31 Sino-Japanese football policies, the study finds that the high-frequency words in China's 18 policies are dominated by "campus, construction, management, reform", which reflects China’s emphasis on football popularization and adopts a government-led, top-down governance logic. The high-frequency words in Japan's 13 policies are dominated by "JFA, players, coaches, leagues, World Cup", which reflects Japan's emphasis on the competitive level of football and adopts an association-led, market-driven, competition-centered development logic. In the analysis of policy instrument mix, 6 football policies respectively from China and Japan were selected for analysis.

We have added the following content on page 25, lines 650-651: Among the 12 Sino-Japanese football policies analyzed using the PMC index model,

We have reviewed, revised and standardized the reference formatting throughout the manuscript, and corrected the URLs for references 2 and 3.

We have carefully addressed all the comments and suggestions you provided point by point. A detailed point-by-point response letter is attached, in which we have responded to each comment individually and clearly indicated the corresponding revisions in the manuscript (with page and line numbers). We believe that these revisions have addressed all the concerns raised and have further strengthened the academic value of this paper.

If you have any further questions or require additional revisions, please do not hesitate to contact us. We look forward to your positive response and sincerely hope that our manuscript will be accepted for publication in your esteemed journal.
