# Supplementary material for: The comparative analysis of Sino-Japanese football policies from the perspective of policy instruments
Source: PLoS One. 2026 Jul 29;21(7):e0354667. doi: 10.1371/journal.pone.0354667 (PMC13421760; doi:10.1371/journal.pone.0354667)
Supplement: Peer review history

# Peer review: Decision Letter 0

**DOI:** 10.1371/journal.pone.0354667.r001

**Authors:** Mario Lopes

10 Feb 2026

PONE-D-25-49612

The Comparative Analysis of Sino-Japanese Football Policies from the Perspective of Policy Tools

PLOS One

Dear Dr. Zhang,

Thank you for submitting your manuscript to PLOS ONE. After careful consideration, we feel that it has merit but does not fully meet PLOS ONE’s publication criteria as it currently stands. Therefore, we invite you to submit a revised version of the manuscript that addresses the points raised during the review process.

Both reviewers value the paper’s originality, structure, and methodological rigor, recognizing its contribution to comparative sports policy research. They recommend improving the English for clarity, simplifying the abstract and conclusion, and separating the discussion from final remarks. The introduction should better contextualize Sino‑Japanese football policies within broader international frameworks, while the analysis should critically explore whether “Policy Clarity” issues stem from deliberate strategy or governance limits. Reviewers also suggest clarifying the added value of visualizations, deepening the discussion of structural differences between the two countries, and offering more concrete, actionable examples in the policy recommendations.

Please submit your revised manuscript by the Mar 27 2026 11:59PM. If you will need more time than this to complete your revisions, please reply to this message or contact the journal office at plosone@plos.org. When you're ready to submit your revision, log on to https://www.editorialmanager.com/pone/ and select the 'Submissions Needing Revision' folder to locate your manuscript file.

Please include the following items when submitting your revised manuscript:

- A letter that responds to each point raised by the academic editor and reviewer(s). You should upload this letter as a separate file labeled 'Response to Reviewers'.
- A marked-up copy of your manuscript that highlights changes made to the original version. You should upload this as a separate file labeled 'Revised Manuscript with Track Changes'.
- An unmarked version of your revised paper without tracked changes. You should upload this as a separate file labeled 'Manuscript'.

If you would like to make changes to your financial disclosure, please include your updated statement in your cover letter. Guidelines for resubmitting your figure files are available below the reviewer comments at the end of this letter.

If applicable, we recommend that you deposit your laboratory protocols in protocols.io to enhance the reproducibility of your results. Protocols.io assigns your protocol its own identifier (DOI) so that it can be cited independently in the future. For instructions see: https://journals.plos.org/plosone/s/submission-guidelines#loc-laboratory-protocols. Additionally, PLOS ONE offers an option for publishing peer-reviewed Lab Protocol articles, which describe protocols hosted on protocols.io. Read more information on sharing protocols at https://plos.org/protocols?utm_medium=editorial-email&utm_source=authorletters&utm_campaign=protocols.

We look forward to receiving your revised manuscript.

Kind regards,

Mario Lopes, Ph.D

Academic Editor

PLOS One

Journal Requirements:

When submitting your revision, we need you to address these additional requirements.

1. Please ensure that your manuscript meets PLOS ONE’s style requirements, including those for file naming. The PLOS ONE style templates can be found at

https://journals.plos.org/plosone/s/file?id=wjVg/PLOSOne_formatting_sample_main_body.pdf and

https://journals.plos.org/plosone/s/file?id=ba62/PLOSOne_formatting_sample_title_authors_affiliations.pdf

2. We note that the grant information you provided in the ‘Funding Information’ and ‘Financial Disclosure’ sections do not match.

When you resubmit, please ensure that you provide the correct grant numbers for the awards you received for your study in the ‘Funding Information’ section.

3. Thank you for stating the following financial disclosure:

“Chinese Social Science Foundation Project (23BTY045)and the Major Social Science Research Project of Tianjin Municipal Education Commission (2022JWZD57).”

Please state what role the funders took in the study.  If the funders had no role, please state: "The funders had no role in study design, data collection and analysis, decision to publish, or preparation of the manuscript."

If this statement is not correct you must amend it as needed.

Please include this amended Role of Funder statement in your cover letter; we will change the online submission form on your behalf.

4. We note that your Data Availability Statement is currently as follows: “All relevant data are within the manuscript and its Supporting Information files.”

Please confirm at this time whether or not your submission contains all raw data required to replicate the results of your study. Authors must share the “minimal data set” for their submission. PLOS defines the minimal data set to consist of the data required to replicate all study findings reported in the article, as well as related metadata and methods (https://journals.plos.org/plosone/s/data-availability#loc-minimal-data-set-definition).

For example, authors should submit the following data:

- The values behind the means, standard deviations and other measures reported;

- The values used to build graphs;

- The points extracted from images for analysis.

Authors do not need to submit their entire data set if only a portion of the data was used in the reported study.

If your submission does not contain these data, please either upload them as Supporting Information files or deposit them to a stable, public repository and provide us with the relevant URLs, DOIs, or accession numbers. For a list of recommended repositories, please see https://journals.plos.org/plosone/s/recommended-repositories.

If there are ethical or legal restrictions on sharing a de-identified data set, please explain them in detail (e.g., data contain potentially sensitive information, data are owned by a third-party organization, etc.) and who has imposed them (e.g., an ethics committee). Please also provide contact information for a data access committee, ethics committee, or other institutional body to which data requests may be sent. If data are owned by a third party, please indicate how others may request data access.

5. Please include a separate caption for each figure in your manuscript.

6. If the reviewer comments include a recommendation to cite specific previously published works, please review and evaluate these publications to determine whether they are relevant and should be cited. There is no requirement to cite these works unless the editor has indicated otherwise.

Additional Editor Comments:

Both reviewers appreciate the paper’s originality and methodological rigor, recognizing its strong structure and potential contribution to comparative sports policy research. However, they also point out several areas for improvement to enhance readability, coherence, and critical depth. The English language and overall presentation need careful revision to ensure clarity and fluency, particularly in the abstract and throughout the text. The introduction should be expanded to situate Sino‑Japanese football policies within a wider international and Asian context, establishing a stronger comparative foundation. Reviewers suggest that the analysis could go beyond descriptive reporting by critically examining whether the observed weakness in “Policy Clarity” reflects deliberate strategic ambiguity or broader governance limitations, especially within the Chinese context. Although the visualizations are technically impressive, their analytical value should be clarified or streamlined to avoid redundancy. The conclusion section should be restructured into a concise, cohesive synthesis that separates discussion from closing insights and offers clear, actionable recommendations. Strengthening the discussion of governance differences between China and Japan, including how institutional structures shape policy implementation, will add needed depth, as will more precise examples of stakeholder diversification and youth‑coach‑team integration. Finally, acknowledging that football remains less culturally dominant despite strong policy efforts would enrich the contextual interpretation. Together, these revisions will help transform the current manuscript into a clearer, more engaging, and analytically powerful contribution to international sports policy scholarship.

[Note: HTML markup is below. Please do not edit.]

Reviewers' comments:

Reviewer’s Responses to Questions

**Comments to the Author**

1. Is the manuscript technically sound, and do the data support the conclusions?

The manuscript must describe a technically sound piece of scientific research with data that supports the conclusions. Experiments must have been conducted rigorously, with appropriate controls, replication, and sample sizes. The conclusions must be drawn appropriately based on the data presented.

Reviewer #1: Yes

Reviewer #2: Yes

**********

2. Has the statistical analysis been performed appropriately and rigorously?

Reviewer #1: I Don't Know

Reviewer #2: Yes

**********

3. Have the authors made all data underlying the findings in their manuscript fully available?

The PLOS Data policy requires authors to make all data underlying the findings described in their manuscript fully available without restriction, with rare exception (please refer to the Data Availability Statement in the manuscript PDF file). The data should be provided as part of the manuscript or its supporting information, or deposited to a public repository. For example, in addition to summary statistics, the data points behind means, medians and variance measures should be available. If there are restrictions on publicly sharing data—e.g. participant privacy or use of data from a third party—those must be specified.

Reviewer #1: Yes

Reviewer #2: Yes

**********

4. Is the manuscript presented in an intelligible fashion and written in standard English?

PLOS ONE does not copyedit accepted manuscripts, so the language in submitted articles must be clear, correct, and unambiguous. Any typographical or grammatical errors should be corrected at revision, so please note any specific errors here.

Reviewer #1: No

Reviewer #2: Yes

**********

5. Review Comments to the Author

Please use the space provided to explain your answers to the questions above. You may also include additional comments for the author, including concerns about dual publication, research ethics, or publication ethics. (Please upload your review as an attachment if it exceeds 20,000 characters)

Reviewer #1: I find the article interesting and thought-provoking in terms of sports policy, specifically soccer policy in two countries with little history or tradition in this sport.

The comments are intended to help the authors improve their proposal:

1. The English in the article needs improvement;

2. The summary is confusing;

3. Not only football policies can be a factor in change and sports development, but sports policies in general. If only football needs to be further exemplified;

4. The methodological proposal seems innovative. On the other hand, there is a lot of information and the reader gets lost in the quantity and complexity of the data presented. We suggest that the authors develop a more intuitive data presentation proposal that actually compares the policies of different countries;

5. The conclusion in bullet points provides what one would expect from a discussion of the results, leaving out an actual conclusion of the text.

Authors will ultimately face the challenge of making the text more enjoyable and effective for readers. Good luck.

Reviewer #2: Thank you for the opportunity to revise this paper. I must state that, I do NOT have a conflict of interest to declare.

Overall opinion of this referee: This paper provides a structured and detailed analysis of Sino-Japanese football policies from the perspective of policy tools. The study is well-organized, with clear sections: introduction, research design, analysis, quantitative evaluation, conclusions, and policy implications. It uses established methodologies, such as content analysis, the PMC index model, and quantitative evaluation, to assess the policies' strengths and weaknesses. The findings are presented systematically, supported by tables, figures, and references, and the conclusions are followed by actionable policy recommendations. While the paper acknowledges some limitations, it offers a comprehensive and logical approach to the topic.

Some suggestions to improve the quality of the paper.

The authors could strengthen the introduction by examining football policies more broadly, worldwide, including in Asia and neighboring countries, and then exploring the policies of China and Japan.

The PMC results show that Policy Clarity is a consistent weakness for both nations. However, the paper fails to critically examine whether this lack of clarity is a strategic choice or a governance failure, particularly in the Chinese context, where macro-level direction is often prioritized over operational details.

While the MATLAB-generated surface plots are visually impressive, they largely confirm what is already in the tables. The paper does not sufficiently explain how these visualizations provide a unique analytical advantage over raw PMC index scores.

The Conclusion section is too long and does not constitute a genuine scholastic paper conclusion. The conclusion section should succinctly summarize the key findings, highlight the main takeaways, and provide actionable recommendations for policy improvement. This factual separation from the discussion would make the paper more organized and ensure that the conclusions are presented in a focused and impactful manner, in a short, coherent, and meaningful paragraph.

The authors should discuss the structural differences between China’s government model and Japan’s model. Explaining how these different issuing authorities can affect policy implementation would add significant depth.

The recommendation to promote stakeholder diversification is vague. The paper should provide concrete examples of how China could adopt Japan’s three-pronged approach (Coach, youth, and National team cohesiveness) within a specific regulatory framework.

While the paper identifies that China lacks focus on World Cup participation targets in its policies compared to Japan (the author may put the results of the Japan team in the previous World Cups), it should explore why (e.g., differing philosophies of long-term vs short-term success) to provide more actionable ‘international benchmarking.

The authors could add to the Discussion that football is neither the most common nor the preferred sport or physical activity among children and students, despite strong government policies.

**********

6. PLOS authors have the option to publish the peer review history of their article (what does this mean?). If published, this will include your full peer review and any attached files.

If you choose “no”, your identity will remain anonymous but your review may still be made public.

**Do you want your identity to be public for this peer review?**  For information about this choice, including consent withdrawal, please see our Privacy Policy.

Reviewer #1: No

Reviewer #2: No

**********

[NOTE: If reviewer comments were submitted as an attachment file, they will be attached to this email and accessible via the submission site. Please log into your account, locate the manuscript record, and check for the action link "View Attachments". If this link does not appear, there are no attachment files.]

To ensure your figures meet our technical requirements, please review our figure guidelines: https://journals.plos.org/plosone/s/figures

You may also use PLOS’s free figure tool, NAAS, to help you prepare publication quality figures: https://journals.plos.org/plosone/s/figures#loc-tools-for-figure-preparation.

NAAS will assess whether your figures meet our technical requirements by comparing each figure against our figure specifications.

# Author comment: Author response to Decision Letter 1

**DOI:** 10.1371/journal.pone.0354667.r002

30 Mar 2026

We appreciate the valuable comments and suggestions from the editor and reviewers. We have revised the manuscript accordingly.

Reviewer #1:

1. The English in the article needs improvement;

Response: Thank you for your reminder, Reviewer. We have carefully optimized and improved the English expression throughout the manuscript, corrected the non-standard and incoherent expressions, and strived to make the English expression accurate, rigorous and fluent to better present the research content.

2. The summary is confusing;

Response: We fully agree with your judgment. We have completely rewritten, revised and improved the abstract section, sorted out the logical context, optimized the language expression, to ensure that the core viewpoints of the abstract are clear, logically coherent and hierarchical, and accurately summarize the research focus and core conclusions of the entire manuscript.

3. Not only football policies can be a factor in change and sports development, but sports policies in general. If only football needs to be further exemplified;

Response: Thank you for your professional advice. We have deleted the statement "football policies play an important role in football reform and development" in the abstract to effectively avoid ambiguity, ensure more rigorous expression, and conform to your guiding opinions.

4. The methodological proposal seems innovative. On the other hand, there is a lot of information and the reader gets lost in the quantity and complexity of the data presented. We suggest that the authors develop a more intuitive data presentation proposal that actually compares the policies of different countries;

Response: We sincerely thank you for your suggestion. We have simplified the relevant descriptions in Section 3.2.4 of the manuscript. Regarding the data presentation form, presenting word frequency, multi-input-output scale and PMC index table in the form of tables is a conventional method in PMC policy analysis. For example, in the following papers: 1. Hong S, Wang T, Fu X, Li G. Research on quantitative evaluation of digital economy policy in China based on the PMC index model. Peng J, editor. PLOS ONE. 2024;19: e0298312. doi:10.1371/journal.pone.0298312; 2. Cui C, Wang K. Quantitatively analyzing the college student employment policy in China based on PMC-index model. Cobo-Martín MJ, editor. PLOS ONE. 2024;19: e0310479. doi:10.1371/journal.pone.0310479, such table forms are used. Therefore, this form is still retained in this revision, and we sincerely request your understanding.

5. The conclusion in bullet points provides what one would expect from a discussion of the results, leaving out an actual conclusion of the text.

Response: We deeply recognize the importance of this issue. We have completely rewritten and revised the conclusion and suggestion sections, making the conclusion section more concise and concise, highlighting the key points, and fully reflecting the summarization and generality of the entire research; at the same time, we have optimized the suggestion section, closely combining the research results and core findings of the manuscript to enhance the pertinence and relevance of the suggestions, ensuring that they meet the normative requirements of conclusions and suggestions in academic papers.

We would like to express our sincere gratitude to you again for your careful guidance and professional advice. We have made corresponding revisions in accordance with all the suggestions you put forward. Although we have strived for perfection, there may still be omissions. We sincerely request you to give further corrections. We will attach great importance to your subsequent opinions, promptly and earnestly make improvements and revisions, and make every effort to improve the quality of the manuscript, living up to your guidance and expectations.

Reviewer #2:

1.The authors could strengthen the introduction by examining football policies more broadly, worldwide, including in Asia and neighboring countries, and then exploring the policies of China and Japan.

Response: Thank you for your valuable suggestion. We have added relevant literature in the introduction to broaden the research perspective, including: the impact of anti-hooligan policies on football match ticket sales, football club transfer policies, the impact of policies prohibiting the sale of alcoholic beverages in football stadiums on crime rates, the impact of economic control policies on the finances of professional football clubs, and the impact of football policies on football match safety.

2.The PMC results show that Policy Clarity is a consistent weakness for both nations. However, the paper fails to critically examine whether this lack of clarity is a strategic choice or a governance failure, particularly in the Chinese context, where macro-level direction is often prioritized over operational details.

Response: We deeply agree with your judgment. We have added relevant content in Section 4.5.1 of the manuscript: "As indicated in mean values of primary variables, both China and Japan achieved low values in policy clarity. In China’s case, inadequate policy clarity was mainly ascribed to the top-down design and hierarchical implementation of its sports policies, which tended to result in ambiguous definitions of specific details. For Japan, the absence of policy clarity in football policies was attributed to its market-led policy framework, so that, in formulating the policies, it cannot better balance the demands of multiple entities, including football associations, professional league bodies, clubs, and local governments, thereby resulting in ambiguous policy statements."

3.While the MATLAB-generated surface plots are visually impressive, they largely confirm what is already in the tables. The paper does not sufficiently explain how these visualizations provide a unique analytical advantage over raw PMC index scores.

Response: Thank you for your professional suggestion. We have added " The PMC surface plot can matrix the first-level variable values of each policy, and the advantages and disadvantages of the policy can be directly observed through the surface plot." in Section 4.4 of the manuscript, and added the following content in Section 4.5.2: " As illustrated by Table 8 and Figure 1, CP3 yields the highest PMC index among Sino-Japanese football policies."; " It can be seen from Figure 2 that the policy exhibits a relatively significant depression in X8 (Policy Clarity)."; " It can be seen from Figure 3 that JP2 shows certain depressions in X2 (Policy Timeframe) and X9 (Policy Targets), indicating that the policy not only lacks short-term and ultra-short-term plans but also has an unclear division of responsibilities between government departments and clubs."; " It can be seen from Figure 4 that the policy has obvious deficiencies in X2 (Policy Timeframe), X4 (Policy Objectives), X6 (Policy Instruments) and X8 (Policy Clarity), because the policy ignores specific implementation plans and details."

4.The Conclusion section is too long and does not constitute a genuine scholastic paper conclusion. The conclusion section should succinctly summarize the key findings, highlight the main takeaways, and provide actionable recommendations for policy improvement. This factual separation from the discussion would make the paper more organized and ensure that the conclusions are presented in a focused and impactful manner, in a short, coherent, and meaningful paragraph.

Response: Thank you for your careful guidance. We have improved the relevant content in strict accordance with your requirements. On the one hand, we have targeted deleted and refined the conclusion section, abandoned redundant expressions, briefly and concisely summarized the main research findings of the entire manuscript, highlighted the core points, and strived to present the conclusion in a short, coherent and powerful paragraph; on the other hand, we have revised and improved the suggestion section, put forward executable policy improvement suggestions focusing on the core research findings, and clearly distinguished the content boundary between the conclusion and the discussion section, further sorted out the manuscript structure, to ensure that the logic of the manuscript is clearer, the expression is more rigorous, and it conforms to the normative requirements of the conclusion of an academic paper.

5.The authors should discuss the structural differences between China’s government model and Japan’s model. Explaining how these different issuing authorities can affect policy implementation would add significant depth.

Response: Thank you for your careful review and professional advice. We have added relevant content in Part 1 of the suggestions: Football policies promulgated by JFA are characterized by their practice orientation and flexible adaptation, but are lacking in adequate administrative binding force in resource coordination and cross-domain advancement, resulting in sluggish policy implementation. China’s football policies are issued by the government departments. Their advantages are embodied in their strong administrative coordination and resource allocation capabilities, while their disadvantage is the great tendency to lead to a one-size-fits-all situation.

6.The recommendation to promote stakeholder diversification is vague. The paper should provide concrete examples of how China could adopt Japan’s three-pronged approach (Coach, youth, and National team cohesiveness) within a specific regulatory framework.

Response: Thank you for your professional suggestion. We have added relevant content in Part 3 of the suggestions: Through incorporating Japanese strategies of cultivating and assessing coaches, we can progressively nurture female coaches from sports colleges and grassroots sectors, constantly increase the number of registered coaches, and enhance their teaching capacity and competence through continuing education and professional training. Notwithstanding China’s introduction of many robust supportive policies from the state level, football is not the most widespread and the most preferred sport among children and youth. Accordingly, in terms of youth’s training, we should draw on Japan’s football model of equal emphasis on popularization and elite to advance the youth training system that integrates campuses, clubs and local football associations. In terms of the cohesiveness of national teams, we should learn from Japanese culture-led strategies to positively publicize national teams’ competitive spirit, reinforce their ideological and cultural construction, and foster their spirit of solidarity, striving and unyieldingness, thus collectively promoting the sound, scientific and sustainable development of Chinese football cause.

7.While the paper identifies that China lacks focus on World Cup participation targets in its policies compared to Japan (the author may put the results of the Japan team in the previous World Cups), it should explore why (e.g., differing philosophies of long-term vs short-term success) to provide more actionable ‘international benchmarking.

Response: Thank you for your suggestion. We have added the following content in Section 3.5.1: Japan Men’s National Football Team has qualified for seven consecutive World Cups since their debut in the 1998 tournament in France, and progressed beyond the group stage on four occasions—in 2002, 2010, 2018, and 2022. Thereby, five of Japan’s six football policies explicitly mentioned World Cup participation objectives.

We have added the following content in Part 2 of the suggestions: Japan’s football policies consistently take claiming the World Cup as their long-term development objective, while China’s football policies lack the long-term planning objective for participating in the World Cup.

8.The authors could add to the Discussion that football is neither the most common nor the preferred sport or physical activity among children and students, despite strong government policies.

Response: Thank you again for your careful guidance. We have added the following content in Part 3 of the suggestions: Although strong support policies have been introduced at the national level, football is not the most popular or favored sport among children and adolescents.

We have revised the relevant suggestions you put forward. If there are any inappropriate parts, we sincerely request further corrections. We will make timely improvements to ensure the research quality.

Attachment Submitted filename: Response to Reviewers.docx

# Editor report: Decision Letter 1

**DOI:** 10.1371/journal.pone.0354667.r003

**Authors:** Mario Lopes

15 Apr 2026

PONE-D-25-49612R1

The Comparative Analysis of Sino-Japanese Football Policies from the Perspective of Policy  Instruments

PLOS One

Dear Dr. Gao,

Thank you for submitting your manuscript to PLOS ONE. After careful consideration, we feel that it has merit but does not fully meet PLOS ONE’s publication criteria as it currently stands. Therefore, we invite you to submit a revised version of the manuscript that addresses the points raised during the review process.-->The manuscript needs clearer concepts, stronger methods, and deeper analysis. The theoretical contribution is not clear enough, as the study is mostly descriptive and does not show how it fits into the wider international sport policy literature. The way policies were chosen is not transparent and could be biased, since terms like “authority” and “relevance” are not clearly defined, and the difference in the number of Chinese and Japanese policies is not explained. Another concern is the use of the PMC index model (pp. 20–22). Using binary scoring and equal weighting makes complex policy content too simple and is not supported by theory, which affects how valid the findings are. The coding process, even though it reports high reliability (p. 16), does not give enough detail about how coding was done or how disagreements were handled. The results are mostly described without much critical analysis or theoretical insight, and some conclusions, especially policy recommendations, seem more like opinions than findings based on the data. The comparison between China and Japan is also not deep enough, as it does not look at important background factors like governance or political systems that could explain the differences. Finally, while the manuscript says all data are available, it does not include the raw coding outputs or NVivo data, which makes it hard to reproduce the study.

Besides these main issues, there are also some smaller problems with clarity and presentation. The manuscript repeats phrases, especially in the abstract and introduction, and some sentences are too long, which makes it harder to read. Some tables, like Table 4 (p. 17), are crowded and not easy to understand. The PMC surface figures (pp. 37–41) are also complex but not well explained in the text, so they do not add much to the analysis. There are a few grammar mistakes and unclear terms that should be fixed for better clarity and consistency. Making the structure clearer, presenting data in a simpler way, and improving the language throughout the manuscript would make it easier to read and improve its quality. Please make a point-by-point response.

Please submit your revised manuscript by May 30, 2026. If you will need more time than this to complete your revisions, please reply to this message or contact the journal office at plosone@plos.org. When you're ready to submit your revision, log on to https://www.editorialmanager.com/pone/ and select the 'Submissions Needing Revision' folder to locate your manuscript file.

Please include the following items when submitting your revised manuscript:

- A letter that responds to each point raised by the academic editor and reviewer(s). You should upload this letter as a separate file labeled 'Response to Reviewers'.
- A marked-up copy of your manuscript that highlights changes made to the original version. You should upload this as a separate file labeled 'Revised Manuscript with Track Changes'.
- An unmarked version of your revised paper without tracked changes. You should upload this as a separate file labeled 'Manuscript'.

If you would like to make changes to your financial disclosure, please include your updated statement in your cover letter. Guidelines for resubmitting your figure files are available below the reviewer comments at the end of this letter.

If applicable, we recommend that you deposit your laboratory protocols in protocols.io to enhance the reproducibility of your results. Protocols.io assigns your protocol its own identifier (DOI) so that it can be cited independently in the future. For instructions see: https://journals.plos.org/plosone/s/submission-guidelines#loc-laboratory-protocols. Additionally, PLOS ONE offers an option for publishing peer-reviewed Lab Protocol articles, which describe protocols hosted on protocols.io. Read more information on sharing protocols at https://plos.org/protocols?utm_medium=editorial-email&utm_source=authorletters&utm_campaign=protocols.

As the corresponding author, your ORCID iD is verified in the submission system and will appear in the published article. PLOS supports the use of ORCID, and we encourage all coauthors to register for an ORCID iD and use it as well. Please encourage your coauthors to verify their ORCID iD within the submission system before final acceptance, as unverified ORCID iDs will not appear in the published article. *Only*  the individual author can complete the verification step; PLOS staff *cannot*  verify ORCID iDs on behalf of authors.

We look forward to receiving your revised manuscript.

Kind regards,

Mario Lopes, Ph.D

Academic Editor

PLOS One

Journal Requirements:

If the reviewer comments include a recommendation to cite specific previously published works, please review and evaluate these publications to determine whether they are relevant and should be cited. There is no requirement to cite these works unless the editor has indicated otherwise.

Additional Editor Comments (if provided):

Your manuscript addresses an important and relevant topic, comparative football policy analysis, and demonstrates a structured approach using policy tools and the PMC index model. The integration of NVivo-based content analysis and quantitative evaluation is a strength. However, several important issues need to be addressed to improve the scientific rigor, clarity, and contribution of the study.

[Note: HTML markup is below. Please do not edit.]

[NOTE: If reviewer comments were submitted as an attachment file, they will be attached to this email and accessible via the submission site. Please log into your account, locate the manuscript record, and check for the action link "View Attachments". If this link does not appear, there are no attachment files.]

To ensure your figures meet our technical requirements, please review our figure guidelines: https://journals.plos.org/plosone/s/figures

You may also use PLOS’s free figure tool, NAAS, to help you prepare publication quality figures: https://journals.plos.org/plosone/s/figures#loc-tools-for-figure-preparation.

NAAS will assess whether your figures meet our technical requirements by comparing each figure against our figure specifications.

# Author comment: Author response to Decision Letter 2

**DOI:** 10.1371/journal.pone.0354667.r004

27 May 2026

Dear Academic Editor,

Thank you for the constructive and detailed comments on our manuscript. We have carefully revised the paper according to each point raised. Below is a point-by-point response, followed by a summary of major revisions made in the manuscript.

1. Reviewer’s comment: The manuscript needs clearer concepts, stronger methods, and deeper analysis. The theoretical contribution is not clear enough, as the study is mostly descriptive and does not show how it fits into the wider international sport policy literature.

Response: We fully agree and have made substantial revisions.

1.1 Added an international literature review in the Introduction (Pages 2). We cited international studies on policy instrument theory and PMC model applications in sports policy evaluation, clearly positioning our work in the international sport policy literature.

1.2 Added a “Theoretical Framework” section (Page 3, 6). We explicitly state that this study is based on policy instrument theory (McDonnell, 1987) and PMC index model (Ruiz Estrada, 2011), and clarify its contribution: the first systematic PMC-based comparison of Sino–Japanese football policies, filling a gap in Asian sport policy research.

2.Reviewer’s comment: The way policies were chosen is not transparent and could be biased, since terms like “authority” and “relevance” are not clearly defined, and the difference in the number of Chinese and Japanese policies is not explained.

Response:

2.1 We have clarified all selection criteria and explained the numerical difference.

Defined policy selection criteria (Page 3):

Authority: Official documents issued by national government agencies or national football associations.

Relevance: Core content on football development/reform/planning.

Timeliness: Policies issued between 2005–2025.

Explained the number difference (Page 3):

2.2 China: 18 policies (multi-departmental, multi-level government-led system).

Japan: 13 policies (JFA-dominated, fewer government documents).

3. Reviewer’s comment: The coding process, even though it reports high reliability (p. 16), does not give enough detail about how coding was done or how disagreements were handled.

Response: We added detailed coding procedures.

4.1Added coding workflow and reliability check (Page 7-8):

Two coders independently coded all texts.

Disagreements were resolved through discussion; unresolved items were arbitrated by a third expert.

Kappa values >0.87 confirmed high reliability.

4. Reviewer’s comment: Another concern is the use of the PMC index model (pp. 20–22). Using binary scoring and equal weighting makes complex policy content too simple and is not supported by theory, which affects how valid the findings are.

Response: We acknowledge the limitation and provided theoretical justification.

�pages 3, 12�, we point out that the PMC model is widely adopted internationally, and that binary assignment weights can ensure objectivity and comparability. Relevant references have also been added as evidence.

5.Reviewer’s comment: The results are mostly described without much critical analysis or theoretical insight, and some conclusions, especially policy recommendations, seem more like opinions than findings based on the data.

Response: We strengthened data-based analysis and evidence-based recommendations.

5.1 All conclusions are now supported by PMC scores, coding frequencies, and indicator values (Pages 21-22).

5.2 Every recommendation is linked to specific data findings (Page 22-23).

6. Reviewer’s comment: The comparison between China and Japan is also not deep enough, as it does not look at important background factors like governance or political systems that could explain the differences.

Response: In the first paragraph of the conclusion�page 22�, we have added an analysis of key contextual factors such as the governance models and political systems of China and Japan.

China: Government-led, multi-level administrative governance.

Japan: JFA-led, market-oriented, community-based governance.

7. Reviewer’s comment: Finally, while the manuscript says all data are available, it does not include the raw coding outputs or NVivo data, which makes it hard to reproduce the study.

Response: We have provided full supplementary materials.

8. Reviewer’s comment: Besides these main issues, there are also some smaller problems with clarity and presentation…

Response: We have revised thoroughly:

We have invited native English-speaking scholars to revise unclear expressions and grammatical issues, correcting grammatical errors, standardizing terminology, and improving readability. Regarding the table issue, we need to state that existing articles on PMC policy analysis all adopt this table format for statistical presentation. This tabular format is a conventional method in PMC policy analysis and has been widely accepted and adopted in the field (Hong S, 2024). Therefore, in this study, we have continued to use this standardized presentation to ensure consistency of methods and comparability of results. We kindly ask the editor for understanding and approval.

Thank you again for your valuable comments. The revised manuscript has been significantly improved in theoretical depth, methodological rigor, data transparency, and readability. We hope the revised version meets the journal’s standards.

Sincerely,

Corresponding Author

Fei Gao

Attachment Submitted filename: Response_to_Reviewers_auresp_2.docx

# Editor report: Decision Letter 2

**DOI:** 10.1371/journal.pone.0354667.r005

**Authors:** Mario Lopes

8 Jun 2026

PONE-D-25-49612R2

The Comparative Analysis of Sino-Japanese Football Policies from the Perspective of Policy   Instruments

PLOS One

Dear Dr. Gao,

Thank you for submitting your manuscript to PLOS ONE. After careful consideration, we feel that it has merit but does not fully meet PLOS ONE’s publication criteria as it currently stands. Therefore, we invite you to submit a revised version of the manuscript that addresses the points raised during the review process.

The manuscript demonstrates potential; however, substantial revision is necessary to resolve concerns regarding transparency, methodological justification, and reproducibility prior to publication. Although the authors' response letter indicates some improvements, several significant issues persist.

Please submit your revised manuscript by the Jul 23 2026 11:59PM. If you will need more time than this to complete your revisions, please reply to this message or contact the journal office at plosone@plos.org. When you're ready to submit your revision, log on to https://www.editorialmanager.com/pone/ and select the 'Submissions Needing Revision' folder to locate your manuscript file.

Please include the following items when submitting your revised manuscript:

- A letter that responds to each point raised by the academic editor and reviewer(s). You should upload this letter as a separate file labeled 'Response to Reviewers'.
- A marked-up copy of your manuscript that highlights changes made to the original version. You should upload this as a separate file labeled 'Revised Manuscript with Track Changes'.
- An unmarked version of your revised paper without tracked changes. You should upload this as a separate file labeled 'Manuscript'.

If you would like to make changes to your financial disclosure, please include your updated statement in your cover letter. Guidelines for resubmitting your figure files are available below the reviewer comments at the end of this letter.

If applicable, we recommend that you deposit your laboratory protocols in protocols.io to enhance the reproducibility of your results. Protocols.io assigns your protocol its own identifier (DOI) so that it can be cited independently in the future. For instructions see: https://journals.plos.org/plosone/s/submission-guidelines#loc-laboratory-protocols. Additionally, PLOS ONE offers an option for publishing peer-reviewed Lab Protocol articles, which describe protocols hosted on protocols.io. Read more information on sharing protocols at https://plos.org/protocols?utm_medium=editorial-email&utm_source=authorletters&utm_campaign=protocols.

As the corresponding author, your ORCID iD is verified in the submission system and will appear in the published article. PLOS supports the use of ORCID, and we encourage all coauthors to register for an ORCID iD and use it as well. Please encourage your coauthors to verify their ORCID iD within the submission system before final acceptance, as unverified ORCID iDs will not appear in the published article. *Only*  the individual author can complete the verification step; PLOS staff *cannot*  verify ORCID iDs on behalf of authors.

We look forward to receiving your revised manuscript.

Kind regards,

Mario Lopes, Ph.D

Academic Editor

PLOS One

Journal Requirements:

If the reviewer comments include a recommendation to cite specific previously published works, please review and evaluate these publications to determine whether they are relevant and should be cited. There is no requirement to cite these works unless the editor has indicated otherwise.

Additional Editor Comments:

Thank you for your revisions and response letter. While the manuscript has improved in several áreas, particularly the addition of international literature, clarification of policy selection criteria, and reporting of coding reliability, significant concerns remain that preclude acceptance in its current form. With the recommended improvements, the study could make a valuable contribution to comparative sport policy research.

Comment 1: Page 2, line 31 and Page 3, line 10.

The authors mention adding a "Theoretical Framework" section in their response to reviewers, but in the manuscript file, this section is not clearly labeled or separated from the introduction. The theoretical basis (McDonnell & Elmore, 1987; Ruiz Estrada, 2011) is mentioned only in passing within the introduction and methods. Create a clear, labeled Section 2.0 "Theoretical Framework" before Section 2.1. Explicitly state: How policy instrument theory (McDonnell) structures your coding scheme (Table 3). Why the PMC index model (Ruiz Estrada) is appropriate for comparing sports policies across different governance systems (China’s state-led vs. Japan’s association-led). Your theoretical contribution: e.g., "This study integrates policy instrument theory with quantitative PMC modeling to explain how governance systems shape policy instrument mixes in non-Western sport contexts."

2. Policy selection criteria remain ambiguous – especially "authority" and "relevance."

Comment 2: Page 3, lines 20-25.

The authors define "Authority" as "official documents from national governments or football associations" and "Relevance" as "core content on football development/reform/planning." However, it is unclear what constitutes "core content" (e.g., minimum percentage of text? specific keywords?). Provide an operational definition for each:

- Authority: List the exact issuing bodies accepted (e.g., State Council, General Administration of Sport of China, JFA, MEXT). Exclude regional or city-level policies.

- Relevance: A policy is "relevant" if the word "football" appears in the title or in at least 20% of the main text. State this explicitly.

Add a flow diagram in the supplementary materials showing the inclusion/exclusion process with numbers.

Comment 3: Page 3, lines 22-25.

The explanation ("China is multi-departmental, Japan is JFA-dominated") is insufficient. This difference could bias the PMC comparison because more policies allow China to potentially score higher on aggregated metrics. Explicitly state that the comparative PMC analysis is only performed on the 12 matched policies (6 from each country) – not all 31. Explain how those 6 were selected from each national set (e.g., by time period, by policy type, by purposive sampling for comparability). Justify why the other 22 policies were excluded from the quantitative model.

Comment 4: Page 7-8, lines 173-183 (section 3.2.2).

The authors report Kappa > 0.87 but do not describe how many disagreements occurred, what specific nodes caused disagreement, the qualifications of the "third expert" who arbitrated. Add a short paragraph or supplementary table:

"A total of 47 coding disagreements occurred across 592 reference points (7.9%). Disagreements were most frequent in the tertiary nodes 'Platform Construction' (n=12) and 'Functional Delineation' (n=9). The third expert was a professor of sport policy with 10 years of experience. All unresolved items were discussed in two consensus meetings."

Comment 5: Page 12, lines 313-315; also page 3, lines 12-14 (response to reviewers).

The authors defend binary scoring as "widely adopted" but do not address whether equal weighting is valid for football policies where some variables (e.g., funding) may be more important than others (e.g., ultra-short-term planning). Add a Limitation sub-section in the Discussion (around page 28-29) stating: "Binary scoring assumes all secondary variables are of equal importance, which may not reflect real-world policy priorities. Future research should explore weighted PMC models based on expert surveys or empirical outcomes (e.g., World Cup qualification)."

Also run a sensitivity analysis (as a supplementary file) where one or two key variables (e.g., X8 Policy Clarity, X4 World Cup objective) are given double weight, and report whether the ranking changes.

Comment 6: Page 22-23, lines 528-557 (recommendations).

The first recommendation ("Optimize policy instrument combinations") refers to China’s 50% capacity-building and 25% authoritative instruments – good. But the second recommendation ("Remedy existing policy shortcomings") mentions "World Cup participation objectives" – however, Table 8 shows none of China’s policies included World Cup goals (X4-4 = 0 for all CPs). The recommendation is correct, but the link to the data is not explicit. Restructure each recommendation as:

- Finding (data): "Table 8 shows China’s X4 (Policy Objectives) mean value is 0.63, with zero policies mentioning World Cup participation (X4-4 = 0)."

"Therefore, China should add explicit World Cup qualification targets to its medium- and long-term plans."

Comment 7: Page 22, lines 517-519.

The authors contrast China’s "government-led, multi-level administrative governance" with Japan’s "JFA-led, market-oriented governance." This is critical context, but it appears only at the end. Move this comparison to a new sub-section 2.4 "Governance Context of Sino-Japanese Football" before presenting results. Then refer back to it when interpreting differences in policy instruments (e.g., why Japan uses fewer authoritative instruments).

Table 1 comment

Table 1 (keyword frequency) is difficult to interpret with no clear column alignment. The table splits "China" and "Japan" columns but the layout is confusing. Many rows are blank or misaligned. Reformat Table 1 as two separate tables (Table 1a for China, Table 1b for Japan) or use a landscape layout with clear vertical lines separating the two countries. Ensure all 40 ranks are visible.

Table 8 comment

A row labeled "Concavity Index" appears with numerical values (2.11, 1.37, etc.) but the concept is never defined in the text. Add a sentence in Section 4.3 or 4.4: "The Concavity Index measures the average deviation of the PMC surface from a flat plane; higher values indicate greater variability among primary variable scores." Alternatively, remove it if not interpreted.

Typographical and grammatical errors comments

Page 1, line 34: "football’s progress": should be "football’s development" (repetitive).

Page 12, line 44: "Overall Plan for Chinese Football Reform and Development [2] (hereinafter referred to as The Plan)": missing closing quotation mark or italics.

Page 29, line 416: "five of Japan’s six football policies": but Japan has 13 total and 6 in the PMC sample. Be specific: "five of the six Japanese policies in the PMC analysis."

Perform a full copy-edit focusing on consistent use of definite/indefinite articles, clear differentiation between "China’s policies" (all 18) vs "the six selected Chinese policies.", standardized reference formatting (some URLs are broken, e.g., ref 2 on page 34).

[Note: HTML markup is below. Please do not edit.]

To ensure your figures meet our technical requirements, please review our figure guidelines: https://journals.plos.org/plosone/s/figures

You may also use PLOS’s free figure tool, NAAS, to help you prepare publication quality figures: https://journals.plos.org/plosone/s/figures#loc-tools-for-figure-preparation.

NAAS will assess whether your figures meet our technical requirements by comparing each figure against our figure specifications.

# Author comment: Author response to Decision Letter 3

**DOI:** 10.1371/journal.pone.0354667.r006

29 Jun 2026

Dear Editor,

Thank you very much for your valuable comments and suggestions on our manuscript. We sincerely appreciate the time and effort you have dedicated to reviewing our research. Your insightful feedback has significantly improved the quality and clarity of the manuscript.

We have carefully addressed all the comments and suggestions you provided point by point. A detailed point-by-point response letter is attached, in which we have responded to each comment individually and clearly indicated the corresponding revisions in the manuscript (with page and line numbers). We believe that these revisions have addressed all the concerns raised and have further strengthened the academic value of this paper.

If you have any further questions or require additional revisions, please do not hesitate to contact us. We look forward to your positive response and sincerely hope that our manuscript will be accepted for publication in your esteemed journal.

Attachment Submitted filename: Response_to_Reviewers_auresp_3.docx

# Editor report: Decision Letter 3

**DOI:** 10.1371/journal.pone.0354667.r007

**Authors:** Mario Lopes

12 Jul 2026

The Comparative Analysis of Sino-Japanese Football Policies from the Perspective of Policy   Instruments

PONE-D-25-49612R3

Dear Dr. Gao,

We’re pleased to inform you that your manuscript has been judged scientifically suitable for publication and will be formally accepted for publication once it meets all outstanding technical requirements.

Within one week, you’ll receive an e-mail detailing the required amendments. When these have been addressed, you’ll receive a formal acceptance letter and your manuscript will be scheduled for publication.

An invoice will be generated when your article is formally accepted. Please note, if your institution has a publishing partnership with PLOS and your article meets the relevant criteria, all or part of your publication costs will be covered. Please make sure your user information is up-to-date by logging into Editorial Manager at Editorial Manager® and clicking the ‘Update My Information' link at the top of the page. For questions related to billing, please contact billing support.

If your institution or institutions have a press office, please notify them about your upcoming paper to help maximize its impact. If they’ll be preparing press materials, please inform our press team as soon as possible -- no later than 48 hours after receiving the formal acceptance. Your manuscript will remain under strict press embargo until 2 pm Eastern Time on the date of publication. For more information, please contact onepress@plos.org.

Kind regards,

Mario Lopes, Ph.D

Academic Editor

PLOS One

Additional Editor Comments (optional):

Dear authors,

I congratulate you on the effort placed in the enhancement of the manuscript.

Reviewers' comments:

# Editor report: Acceptance letter

**DOI:** 10.1371/journal.pone.0354667.r008

**Authors:** Mario Lopes

PONE-D-25-49612R3

PLOS One

Dear Dr. Gao,

I'm pleased to inform you that your manuscript has been deemed suitable for publication in PLOS One. Congratulations! Your manuscript is now being handed over to our production team.

At this stage, our production department will prepare your paper for publication. This includes ensuring the following:

* All references, tables, and figures are properly cited

* All relevant supporting information is included in the manuscript submission,

* There are no issues that prevent the paper from being properly typeset

You will receive further instructions from the production team, including instructions on how to review your proof when it is ready. Please keep in mind that we are working through a large volume of accepted articles, so please give us a few days to review your paper and let you know the next and final steps.

Lastly, if your institution or institutions have a press office, please let them know about your upcoming paper now to help maximize its impact. If they'll be preparing press materials, please inform our press team within the next 48 hours. Your manuscript will remain under strict press embargo until 2 pm Eastern Time on the date of publication. For more information, please contact onepress@plos.org.

You will receive an invoice from PLOS for your publication fee after your manuscript has reached the completed accept phase. If you receive an email requesting payment before acceptance or for any other service, this may be a phishing scheme. Learn how to identify phishing emails and protect your accounts at https://explore.plos.org/phishing.

If we can help with anything else, please email us at customercare@plos.org.

Thank you for submitting your work to PLOS ONE and supporting open access.

Kind regards,

PLOS ONE Editorial Office Staff

on behalf of

Prof. Mario Lopes

Academic Editor

PLOS One
